# Electrochemical and Catalytic Efficiency of Green-Synthesized Fe, Ti, and Mixed Fe–Ti Nanoparticles

**DOI:** 10.3390/molecules31193525

**Published:** 2026-10-03

**Authors:** Shamika P. W. R. Hewage, Collin Byas, Debra Davis, Viviana Salazar, Tony L. Grady, Harshica Fernando

**Affiliations:** Department of Chemistry, Prairie View A&M University, Prairie View, TX 77446, USA; shamika-piumal-wishvajith.hewage@ttu.edu (S.P.W.R.H.); tlgrady@pvamu.edu (T.L.G.)

**Keywords:** green synthesis, iron-titanium nanoparticles, cyclic voltammetry, methyl orange, dye degradation, advanced oxidation, dextrose

## Abstract

Catalysts play a crucial role in many chemical reactions, and the synthesis of green catalysts that are economical, selective, and active is important in addressing environmental pollution, a persistent global problem. This study investigates the electrochemical behavior and catalytic efficiency of green-synthesized iron-titanium (Fe-Ti) nanoparticles (NPs) for dye degradation. Three distinct types of NPs were synthesized using an environmentally friendly green approach with dextrose as the reducing agent: pure Fe NPs, pure Ti NPs, and mixed Fe-Ti NPs. The synthesized NPs were characterized using FTIR, XPS, XRD, SEM, and EDS to determine their structural, morphological, and chemical properties, and their redox behavior was evaluated by cyclic voltammetry (CV). Characterizations identified the Fe component as a mixed-valence, chloride-bearing Fe (oxyhydr)oxide most consistent with an akaganeite-type (β-FeOOH) phase and the Ti component as an amorphous Ti^4+^ titania, with the mixed material containing a minor, well-dispersed Fe (oxyhydr)oxide component that preserves accessible Fe^3+^/Fe^2+^ redox activity within the amorphous titania framework. Cyclic voltammetry studies showed that the mixed Fe-Ti NPs possessed the largest electroactive response, with an enclosed voltammetric area of 22.20 µA·V compared with 18.85 and 14.20 µA·V for the Ti and Fe NPs, together with the narrowest polarization gap, indicating the largest overall electrochemical response. The catalytic performance was evaluated through methyl orange (MO) dye degradation followed by UV-Vis spectroscopy, using hydrogen peroxide (H_2_O_2_) as the oxidant with the NPs acting as heterogeneous catalysts in a Fenton-like advanced oxidation process. The mixed Fe-Ti NPs degraded 77.5% of the dye within 60 min, substantially exceeding the Ti NPs (45.1%), the Fe NPs (30.8%), and the catalyst-free control (3.4%). This enhancement is attributed to the combined action of the two components, in which the amorphous titania framework disperses and stabilizes redox-active Fe^3+^/Fe^2+^ centers, coupling the enhanced electrochemical response directly to the Fenton-like generation of reactive radicals that drive dye degradation. These findings show that green-synthesized mixed Fe-Ti NPs represent a promising, dextrose-mediated approach for environmental remediation, particularly in wastewater treatment involving organic dye pollutants.

## 1. Introduction

Water pollution is a growing global concern, and the rapid expansion of the textile, printing, and dyeing industries is one of its most visible drivers. It has been estimated that roughly 700,000 tons of dyes drawn from about 100,000 commercially available formulations are produced each year, a substantial portion of which is lost to effluent during dyeing and finishing and discharged into aquatic environments [1,2]. Azo dyes, which contain one or more nitrogen-to-nitrogen double bonds and constitute the largest single class of synthetic textile colorants (more than 60% of the total), are of particular concern because of their toxicity, environmental persistence, and resistance to conventional biological and physicochemical treatment [1]. MO, a representative sulfonated azo dye, is highly water-soluble, poorly biodegradable, and recalcitrant to standard treatment, and it is therefore widely used as a model pollutant for evaluating new remediation strategies [1]. Developing effective and sustainable approaches for dye degradation is thus a critical environmental challenge.

Advanced oxidation processes (AOPs) have emerged as a powerful alternative to conventional treatment for these refractory pollutants because they generate highly reactive, non-selective hydroxyl radicals capable of mineralizing complex organic molecules into less harmful products [3]. Fenton and Fenton-like chemistry is among the most studied AOPs, in which iron (Fe) species activate hydrogen peroxide (H_2_O_2_) to produce hydroxyl radicals. Homogeneous Fenton systems, however, operate only within a narrow optimal pH range (typically pH 2.5 to 3.5), lose dissolved catalyst to solution, and generate large volumes of Fe sludge, all of which limit their practical use [3]. Heterogeneous Fe -based Fenton catalysts overcome many of these drawbacks by immobilizing the active Fe in a solid phase, which allows operation over a wider pH range, straightforward catalyst recovery and reuse, and minimal secondary contamination [3]. Recent advanced-oxidation research has broadened the routes to hydroxyl (OH)-radical generation, for example through the selective three-electron oxygen reduction reaction that converts O_2_ directly to OH radicals while bypassing the mass-transfer and stability limitations of bulk H_2_O_2_ [4]. A recurring design principle in this work is the introduction of a second metal center to overcome the sluggish Fe(III)/Fe(II) regeneration that limits conventional Fe Fenton chemistry, as demonstrated for Copper (Cu)-doped goethite (Cu-FeOOH), in which a Cu-Fe redox relay accelerates iron cycling and markedly increases the degradation rate of tetracycline [5]. Mixed-metal catalysts composed of two or more metals or metal oxides have attracted considerable attention in this context because they combine synergistic effects, enhanced redox properties, and superior catalytic performance relative to single-metal systems, facilitating degradation pathways such as photocatalysis, Fenton-like oxidation, and persulfate activation [6].

Among these, iron-titanium (Fe-Ti) mixed-metal catalysts have proven highly effective for environmental remediation, particularly for degrading organic dyes and other pollutants in wastewater. The combination integrates the photocatalytic properties of titanium dioxide (TiO_2_) with the strong redox activity of Fe, giving catalysts with improved performance, stability, and sustainability [6,7]. Titanium dioxide is a well-known semiconductor catalyst recognized for its high stability, non-toxicity, and strong oxidative ability under ultraviolet or visible light. Nonetheless, pure TiO_2_ has inherent limitations, including a wide band gap of approximately 3.2 eV and rapid electron-hole recombination, so that it absorbs only about 4 to 5% of the solar spectrum and performs poorly under natural sunlight [6,7]. Incorporating Fe into TiO_2_ helps to overcome these drawbacks by forming Fe-Ti mixed oxides or heterojunctions that narrow the effective band gap, enhance visible-light absorption, promote charge separation, and introduce additional redox sites for generating reactive oxygen species such as OH and superoxide radicals, which play a crucial role in degrading dye molecules and other organic contaminants [7].

Beyond photocatalysis, the Fe-Ti system can also act as a Fenton-like catalyst in which Fe^2+^ and Fe^3+^ ions activate H_2_O_2_ to produce OH radicals. The presence of TiO_2_ enhances the stability of the iron species and supports continuous Fe^2+^/Fe^3+^ redox cycling. This cycling is important because the regeneration of Fe^2+^ from Fe^3+^ is the slow, rate-limiting step of the Fenton process, so any material that accelerates it directly increases the yield of OH radicals and the overall catalytic efficiency [3,8]. This dual functionality, combining photocatalytic and Fenton-like activity, makes Fe-Ti mixed catalysts particularly powerful for water purification [8].

In line with the principles of green chemistry, recent research has focused on eco-friendly synthesis routes for these catalysts. Green Fe-Ti mixed-metal catalysts can be prepared using environmentally benign methods such as sol–gel, hydrothermal, or plant-extract-assisted synthesis, which minimize hazardous chemicals and operate under mild conditions [9]. The use of natural reducing agents such as sugars, together with the avoidance of toxic solvents, further reduces environmental impact, enhances recyclability, and minimizes metal leaching during application [9,10]. In the present work, the term green synthesis is used in this methodological sense. It refers to the replacement of hazardous reductants and stabilizing agents by a benign, renewable reducing sugar (dextrose) under mild, low-temperature, calcination-free conditions, together with the use of ethanol, a low-toxicity and biodegradable solvent. It is not intended as a life-cycle claim regarding the electrical energy used during preparation and measurement or the upstream production of the reagents. In particular, simple reducing sugars such as glucose and dextrose have been shown to act as effective, low-cost, and non-toxic reducing and capping agents for the aqueous synthesis of metal and metal-oxide nanoparticles (NPs), which makes them attractive green precursors for catalyst preparation [9].

A distinguishing feature of the present work is the use of electrochemical characterization by cyclic voltammetry (CV) to rationalize catalytic behavior. For a catalyst intended for advanced oxidation and Fenton-like dye degradation, the redox activity of the material is directly connected to its ability to activate H_2_O_2_ and to sustain radical generation. Cyclic voltammetry reveals the redox couples that are electrochemically accessible in a given material, in particular the Fe^2+^/Fe^3+^ couple that drives the Fenton cycle, along with the reversibility or irreversibility of electron transfer, the onset potentials, and the relative electroactive surface area [11,12]. Recent studies have shown that catalysts exhibiting larger and more reversible redox peaks and a larger electrochemically active surface area also display faster interfacial electron transfer and superior Fenton-like or advanced-oxidation performance toward azo dyes such as Acid Orange 7 [11,12,13]. Correlating these electrochemical descriptors with measured dye-degradation efficiency therefore provides a mechanistic bridge between material characterization and catalytic function. This is precisely the dimension that was not addressed in the group’s earlier study, which focused on lead adsorption rather than on electrochemistry or dye degradation [10]. Table 1 shows a Synthesis comparison of Fe-Ti, Fe and Ti nanoparticles.

Despite extensive work on Fe-Ti and Fe/TiO_2_ materials, important gaps remain. Most green-synthesized Fe-Ti studies report only photocatalytic or adsorption performance; few directly correlate electrochemical redox behavior with catalytic dye-degradation performance for sugar- or dextrose-reduced NPs, and the individual, green-synthesized Fe and Ti NPs are seldom benchmarked side by side against the mixed material under identical conditions [7,10,11]. The present study addresses these gaps and builds directly on the group’s prior demonstration that dextrose-reduced Fe-Ti mixed oxide NPs are effective and sustainable adsorbents for lead removal [10]. The objectives of this work are, in sequence finally synthesize individual Fe, individual Ti, and mixed Fe-Ti NPs by green dextrose reduction under low-temperature, calcination-free conditions; to characterize these materials by Fourier-transform infrared spectroscopy (FTIR), X-ray photoelectron spectroscopy (XPS), X-ray diffraction (XRD), and scanning electron microscopy with energy-dispersive spectroscopy (SEM/EDS); to probe their redox behavior by CV; to compare their catalytic efficiency for MO degradation using H_2_O_2_ as the oxidant in a heterogeneous Fenton-like advanced oxidation process; and finally to relate the observed electrochemical activity to the measured catalytic performance.

## 2. Results

### 2.1. Characterization of Nanoparticles

#### 2.1.1. Fourier-Transform Infrared Spectroscopy

**Figure 1:**
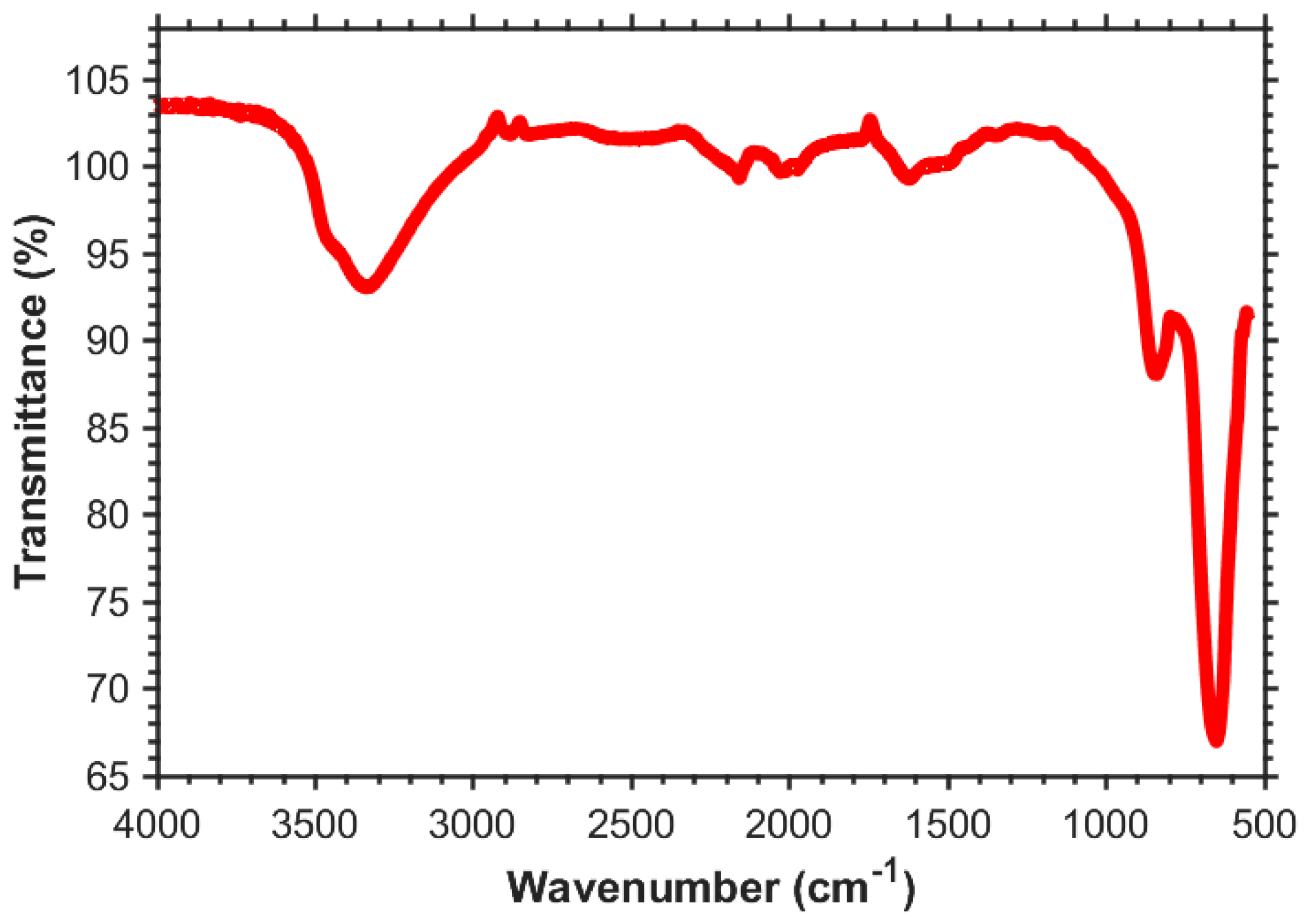
FTIR spectrum of the green-synthesized Fe NPs.

**Figure 2:**
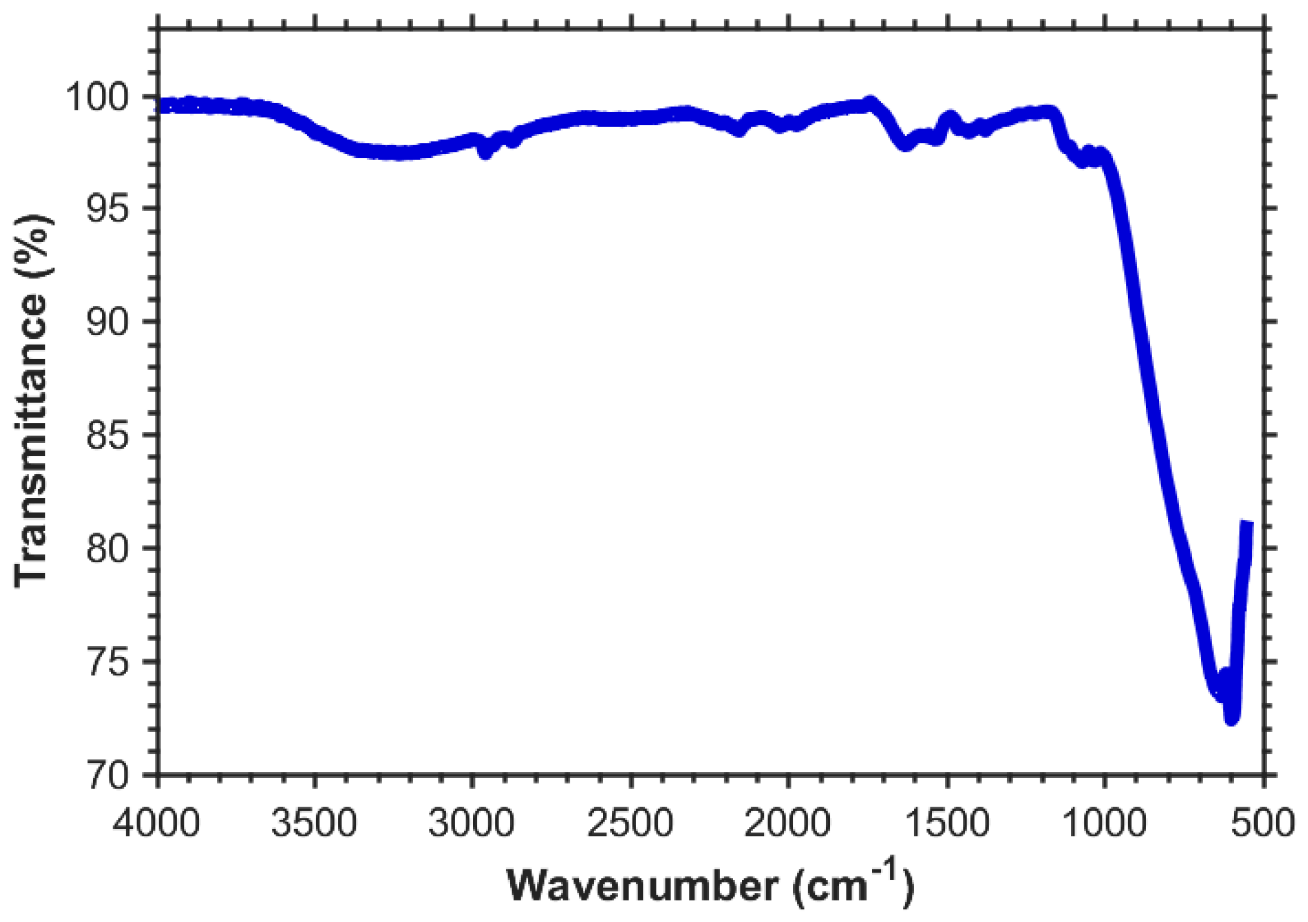
FTIR spectrum of the green-synthesized Ti NPs.

**Figure 3:**
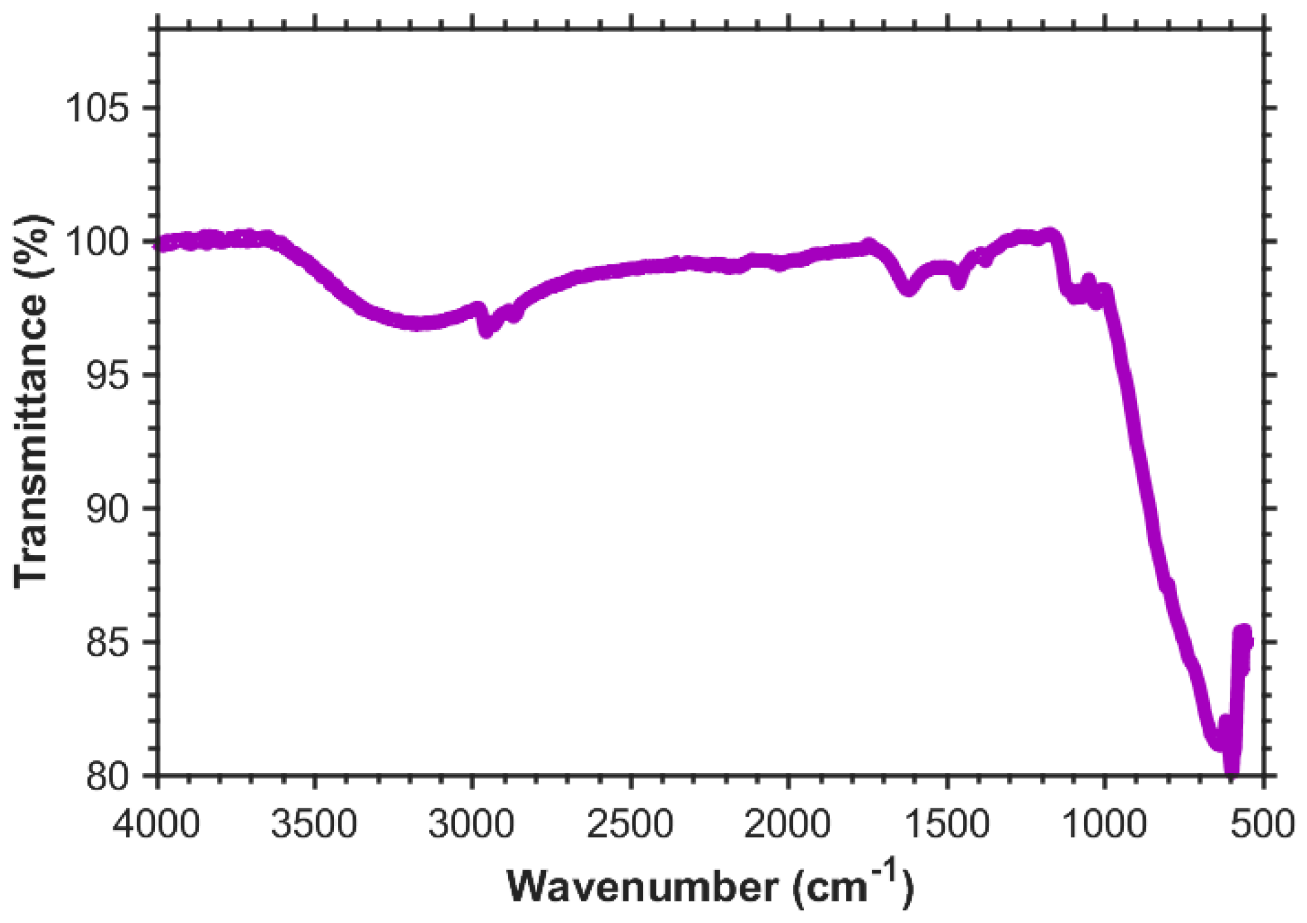
FTIR spectrum of the green-synthesized Fe-Ti NPs.

The surface functional groups of the green-synthesized Fe, Ti, and mixed Fe-Ti NPs were examined by FTIR spectroscopy, following the procedure reported previously for these materials [10]. Spectra were recorded in attenuated total reflectance (ATR) mode on a diamond crystal over the range 4000 to 500 cm^−1^, and a separate measurement was carried out for each of the three samples. Each spectrum was collected in transmittance mode and reported as percent transmittance against wavenumber. FTIR spectroscopy was used to identify the surface functional groups of the three green-synthesized materials and to confirm the formation of the metal oxide network. All three spectra (Figure 1, Figure 2 and Figure 3) share a common set of features, namely a broad OH stretching band in the high-wavenumber region, weak absorptions attributable to residual dextrose-derived organic species, and a strong metal-oxygen (M-O) absorption that dominates the low-wavenumber region and confirms oxide formation in each sample [6,19]. The FTIR spectrum of the Fe NPs displayed a broad band centered at 3341 cm^−1^, assigned to the stretching vibration of surface OH groups, together with a weak band at 1626 cm^−1^ corresponding to the bending mode of O-H and, in part, to carbonyl or carboxylate stretches of residual dextrose-derived organics, and very weak C-H stretching near 2880 cm^−1^ [9,19]. These weak organic features are consistent with a small residual population of dextrose and its oxidation products retained on the particle surface after synthesis, in agreement with the well-established role of reducing sugars as capping and stabilizing agents whose aldehyde groups are oxidized to carboxylates during metal Fe reduction [9]. The most prominent features of the Fe NP spectrum were two well-resolved bands in the low-wavenumber region, an intense band at 653 cm^−1^ assigned to Fe-O stretching and a second band at 843 cm^−1^ assigned to structural Fe-O-H bending, positions that are consistent with the reported infrared signature of the iron oxyhydroxide akaganeite (beta-FeOOH), which shows Fe-O stretching near 650 cm^−1^ and a structural O-H bending doublet near 850 and 820 cm^−1^ [18,20]. The sharpness and low frequency of these bands are characteristic of a crystalline Fe oxyhydroxide rather than a simple Fe oxide, since magnetite, maghemite, and hematite exhibit Fe-O modes at lower wavenumbers, in the approximate range of 430 to 590 cm^−1^ [21]. The FTIR spectrum of the Ti NPs showed a broad, shallow OH stretching band at 3230 cm^−1^ and a weak O-H bending feature at 1629 cm^−1^, indicating surface OH groups and together with weak C-H bands near 2876 and 2962 cm^−1^ and a weak C-O feature at 1073 cm^−1^ from residual organic species [6,22].

The low-wavenumber region was dominated by a strong, broad absorption with minima near 595 and 631 cm^−1^, assigned to the stretching vibrations of the Ti-O and Ti-O-Ti network [22]. The breadth of this envelope and the absence of sharp, well-resolved lattice bands are consistent with a titania network of low structural order formed under the mild, calcination-free synthesis conditions employed here [22]. The FTIR spectrum of the mixed Fe-Ti NPs combined features of the two single-component materials. A broad, weak OH band appeared at 3190 cm^−1^, together with a weak O-H bending and carboxylate feature at 1621 cm^−1^, a weak band at 1463 cm^−1^, and small bands at 1028 and 1097 cm^−1^ attributable to residual C-O functionality [9,10]. The low-wavenumber region again showed a strong M-O absorption, resolved into overlapping minima near 566, 600, and 634 cm^−1^, broader and more complex than the corresponding band of either single oxide, consistent with combined Fe-O and Ti-O contributions within a mixed oxide network [6,16]. The attenuation of the organic C-O and C-H bands, together with the emergence of the strong M-O envelope, confirms the conversion of the dextrose precursor into a metal oxide, reproducing the behavior reported previously for the mixed Fe-Ti material [10]. Across all three samples, the FTIR results confirm the formation of OH-bearing metal oxide networks with only minor residual organic content, and the retention of surface hydroxyl groups is significant because these groups provide reactive sites relevant to the surface chemistry of the materials.

#### 2.1.2. Scanning Electron Microscopy and Energy Dispersive Spectroscopy

**Figure 4:**
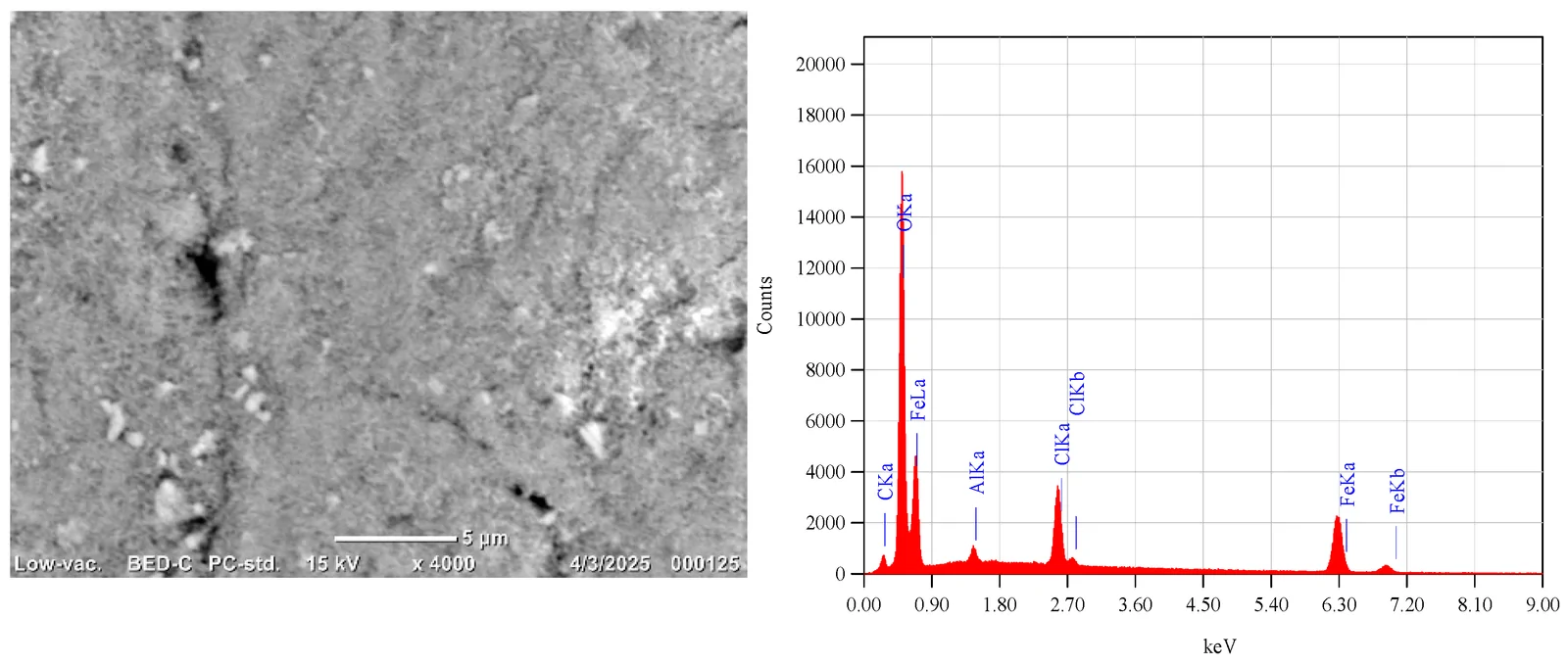
SEM micrograph (**left**) and EDS spectrum (**right**) of the green-synthesized Fe NPs.

The morphology and elemental composition of the green-synthesized NPs were investigated by SEM-EDS. The SEM micrographs revealed distinct surface morphologies for the two individual samples, while the corresponding EDS spectra confirmed their elemental makeup. The SEM micrograph of the Fe NPs (Figure 4), recorded at 4000× magnification, revealed a dense, relatively smooth and continuous surface composed of compact aggregated material, with scattered brighter angular particulates distributed across the surface and a few dark voids.

The compact and aggregated morphology indicates strong particle association during synthesis. The corresponding EDS spectrum confirmed Fe and oxygen (O) as the principal constituents, with an O content of 71.73 atomic percent (50.44 mass percent) and an Fe content of 15.52 atomic percent (38.09 mass percent). The high O level relative to Fe is consistent with the formation of an oxidized Fe phase. Carbon (C) was detected at 7.86 atomic percent, attributable to residual organic species from the dextrose used in the green synthesis, and chlorine (Cl) was present at 4.11 atomic percent (6.41 mass percent), originating from the FeCl_3_ precursor. A minor aluminum signal (0.78 atomic percent) was also recorded. The retention of Cl in the sample indicates that precursor-derived species were incorporated into the material during synthesis.

**Figure 5:**
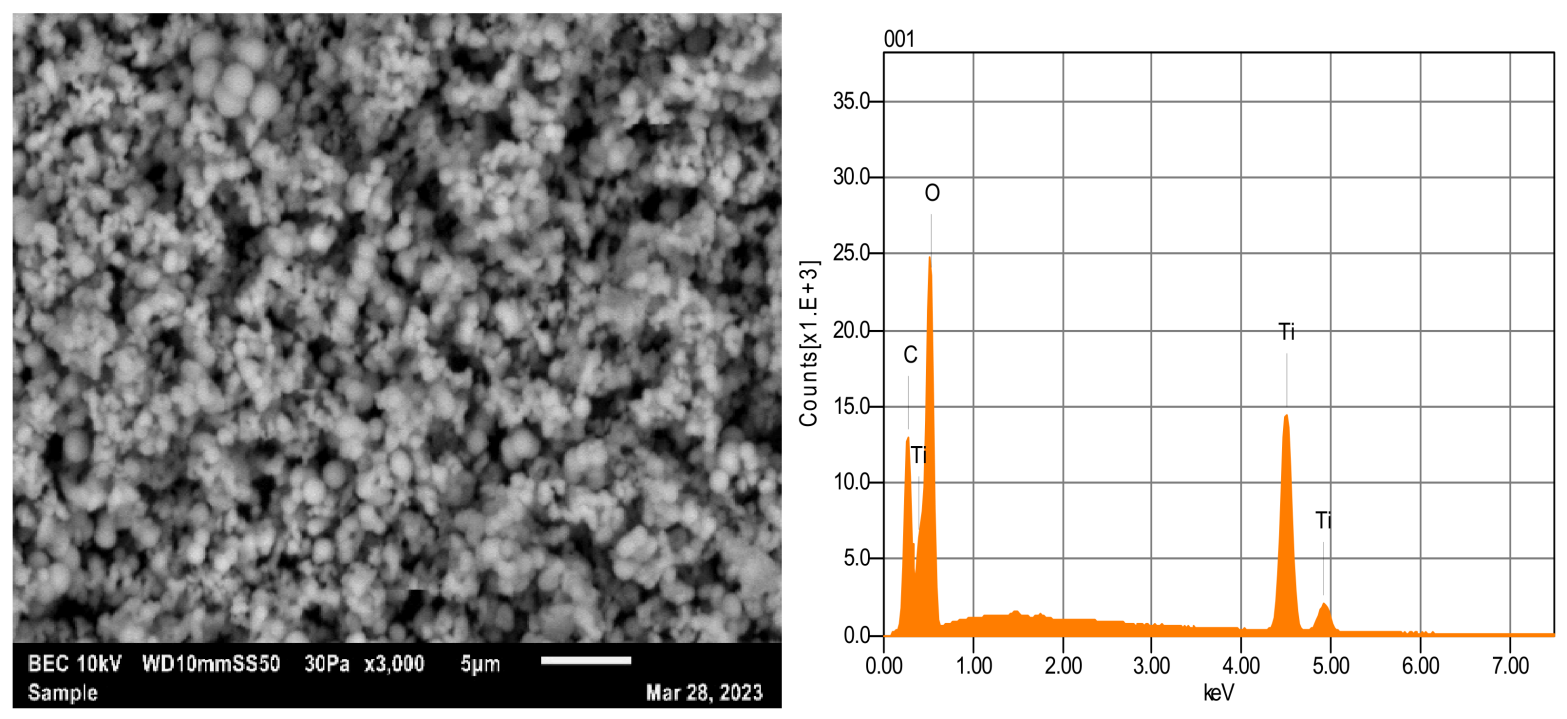
SEM micrograph (**left**) and EDS spectrum (**right**) of the green-synthesized Ti NPs.

The SEM micrograph of the Ti NPs (Figure 5), recorded at 3000× magnification, revealed a markedly different, granular morphology built from densely packed, near-spherical particles with visible interparticle void spaces, giving the surface a more porous and textured appearance than that of the Fe NPs.

This particulate, porous morphology is favorable for providing accessible surface area. The corresponding EDS spectrum confirmed Ti and O as the main elements, with a Ti content of 32.53 atomic percent (61.26 mass percent) and an O content of 43.96 atomic percent (27.64 mass percent), consistent with the formation of TiO_2_. Carbon was detected at 23.51 atomic percent, again attributable to residual dextrose-derived organic species from the green synthesis. No Cl was observed in the Ti NPs, in agreement with the use of a chloride-free Ti precursor. The mixed Fe-Ti NPs [10] previously reported for this system exhibited a heterogeneous surface morphology with irregular aggregates, a rough surface texture, and varied particle sizes forming interconnected structures with visible void spaces between aggregates [10]. Their EDS analysis gave Fe, Ti, O, C, and Cl at atomic percentages of 2.69, 36.03, 43.01, 12.96, and 5.32, respectively, corresponding to a Ti-rich composition with a Ti:Fe atomic ratio of approximately 13:1 [8]. The individual Fe and Ti samples characterized here reproduce the elemental signatures of their respective components within the mixed material, with the Fe NPs contributing Fe and residual Cl and the Ti NPs contributing Ti, and both contributing O and residual synthesis-derived C. The much higher Ti than Fe content of the mixed material is consistent with Ti forming the major framework, and the residual Cl (5.32 atomic percent) parallels the Cl detected in the individual Fe NPs, both originating from the FeCl_3_ precursor.

#### 2.1.3. X-Ray Photoelectron Spectroscopy of Nanoparticles

**Figure 6:**
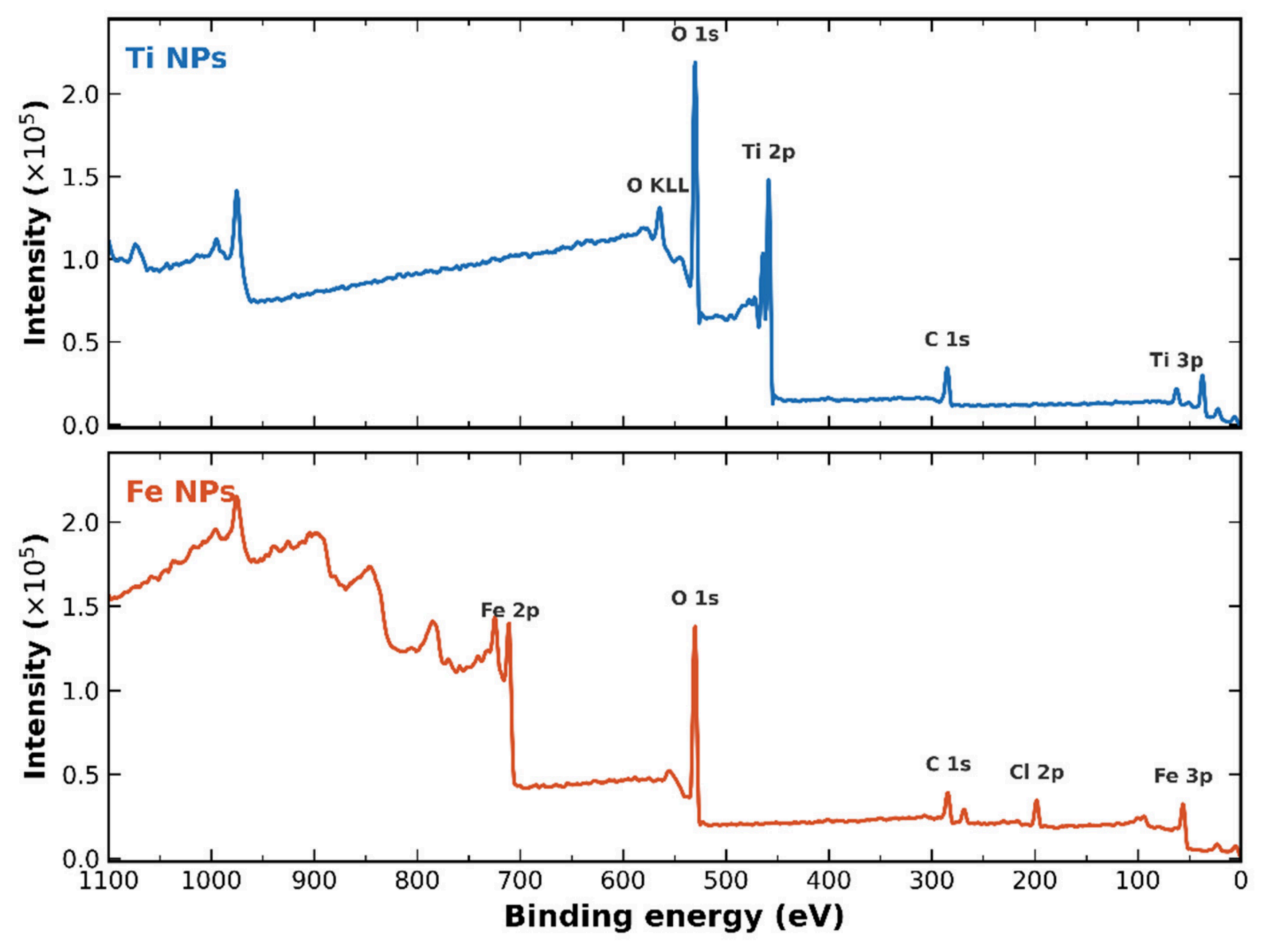
XPS survey scans for the Ti nanoparticle (**top**) and Fe NPs (**bottom**).

The surface chemical composition and electronic states of the green-synthesized Fe, Ti, and mixed Fe-Ti NPs were comprehensively investigated using XPS. The survey spectra of the individual Ti and Fe NPs (Figure 6) revealed the expected surface elements. The Ti NP showed Ti, O, and C with a trace of Cl, with the principal photoelectron and Auger features corresponding to O KLL (~975 eV), Ti 2s (~564 eV), O 1s (~531 eV), Ti 2p (~458 eV), C 1s (~285 eV), and a weak Cl 2p signal (~199 eV). The Fe NP showed Fe, O, C, and Cl, with the main features at O KLL (~975 eV), Fe 2p (~710 eV), O 1s (~531 eV), C 1s (~285 eV), and Cl 2p (~199 eV).

**Figure 7:**
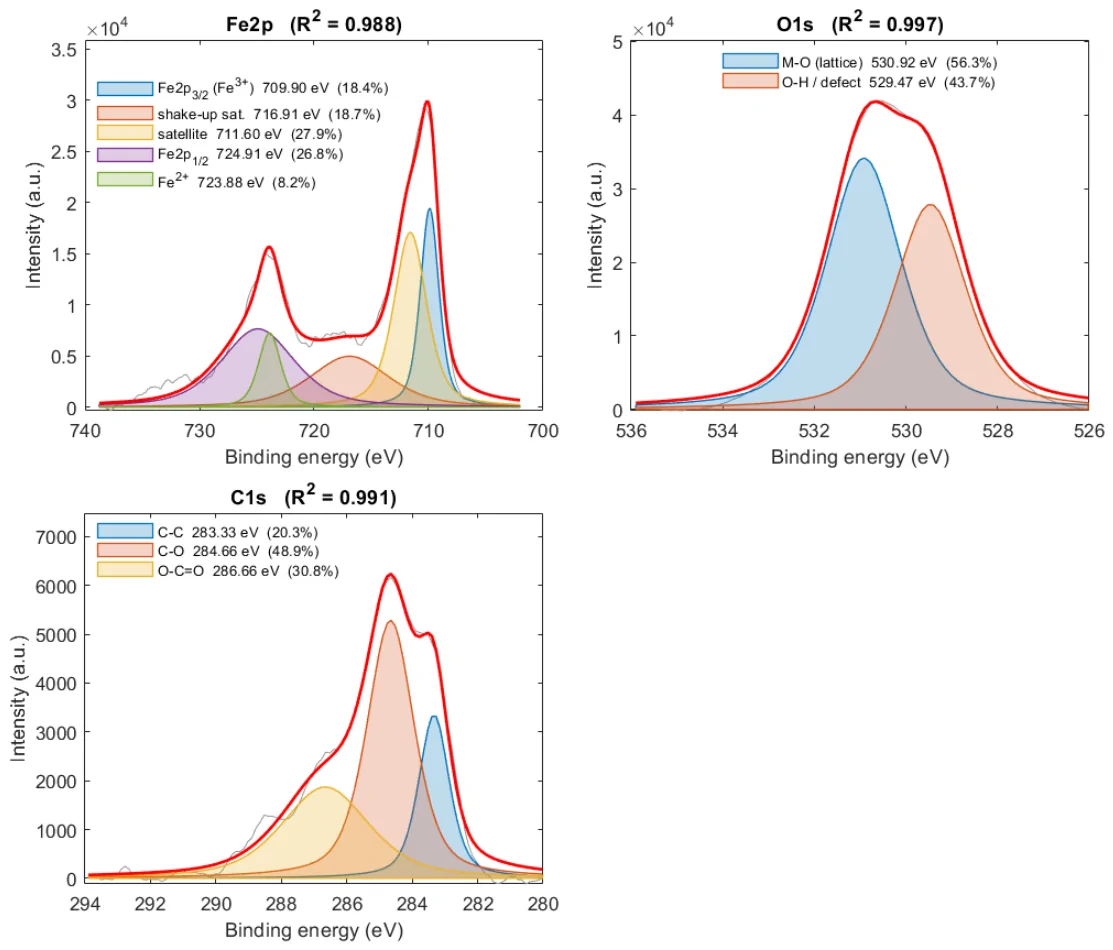
Fe2p (**top left**), O1s (**top right**), and C1s (**bottom left**) deconvoluted high-resolution XPS spectra for the Fe NPs.

**Figure 8:**
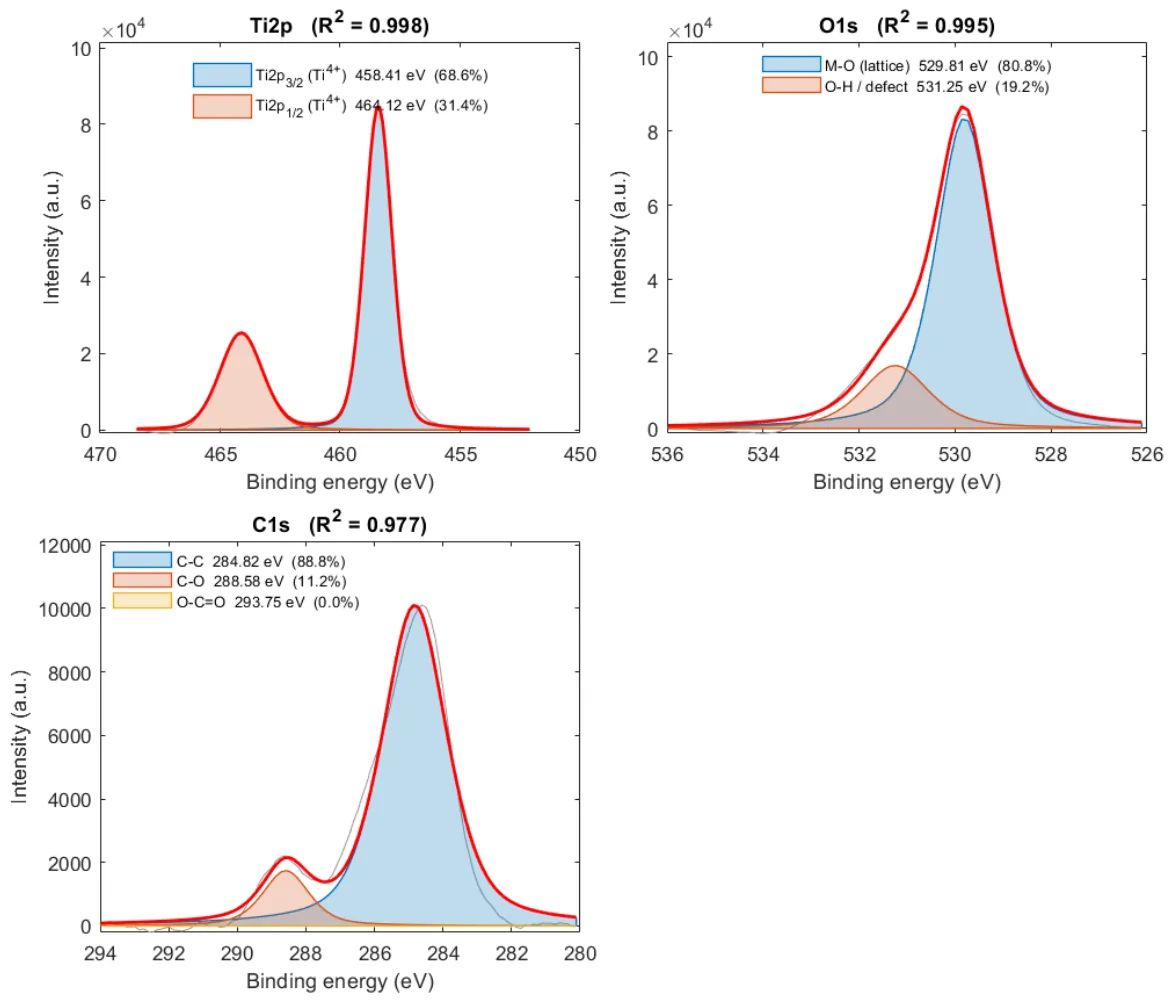
Ti2p (**top left**), O1s (**top right**), and C1s (**bottom left**) deconvoluted high-resolution XPS spectra for the Ti NPs.

For comparison, the survey spectrum of the mixed Fe-Ti NP reported previously revealed Fe, Ti, O, C, and Cl as the main surface elements, with relative atomic percentages of 0.97%, 19.30%, 45.67%, 25.21%, and 8.80%, respectively [10]. In all three samples, the residual Cl is attributed to the metal chloride precursors used in the synthesis, principally FeCl_3_, while the substantial C content reflects adventitious contamination together with residual dextrose-derived organic species retained from the green synthesis route. A detailed chemical state analysis was conducted using high-resolution XPS (Figure 7 and Figure 8).

The C1s spectrum was initially used for charge referencing in each sample, with the adventitious carbon (C-C) peak calibrated to the standard reference value of 284.40 eV for C-C bonds [23], and the resulting offset applied uniformly to all measured binding energies of that sample. For the Ti NP, the C-C peak was observed at 284.82 eV, establishing a negative shift of 0.42 eV, whereas for the Fe NP the C-C peak was observed at 283.33 eV, establishing a positive shift of 1.07 eV. After calibration, the C1s spectrum of the Ti NP resolved into adventitious C-C bonds at 284.40 eV (88.8%) and a higher-binding-energy oxidized-carbon component at 288.16 eV (11.2%) attributable to overlapping C-O and carboxyl (O-C=O) functionalities. The C1s spectrum of the Fe NP revealed three carbon environments: C-C bonds at 284.40 eV (20.3%), C-O bonds at 285.73 eV (48.9%), and carboxyl groups (O-C=O) at 287.73 eV (30.8%). These assignments are consistent with previously reported values for carbon functionalities in metal oxide NPs [23,24], and the pronounced C-O and carboxyl contributions are characteristic of oxygenated organic residues derived from dextrose and its oxidation products remaining on the surface after synthesis [25]. The mixed Fe-Ti NP similarly showed C-C bonds at 284.40 eV (11.36%), C-O bonds at 285.75 eV (10.93%), and carboxyl groups at 288.51 eV (2.92%) [10].

The Fe2p spectrum of the individual Fe NP revealed multiple chemical environments characteristic of mixed-valence Fe-oxide states. After calibration, the primary Fe2p3/2 peak at 709.90 eV shifted to 710.97 eV, corresponding to Fe^3+^ in an oxide environment [26,27], and was accompanied by a higher-binding-energy multiplet shoulder at 712.67 eV arising from the multiplet splitting of high-spin ferric ions and surface oxyhydroxide contributions [28]. A shake-up satellite was observed at 717.98 eV, situated approximately 8 eV above the main 2p3/2 line, a position that is diagnostic of Fe^3+^ because the ferrous (Fe^2+^) shake-up satellite instead appears near 714.7 eV [26,29]. The Fe2p1/2 spin–orbit partner was identified at 724.95 eV, giving a 2p3/2 to 2p1/2 splitting of approximately 14 eV, in agreement with the reported spin–orbit separation of 13.5 to 13.6 eV for Fe^3+^ in Fe oxides [26], with a further contribution at 725.98 eV consistent with mixed-valence character. The position of the main 2p3/2 maximum, intermediate between the reference values of FeO (Fe^2+^, 709.5 eV) and Fe2O3 (Fe^3+^, 711.0 eV) [26], together with the multiplet and satellite structure, indicates a blended Fe^3+^/Fe^2+^ population rather than a single oxidation state. Interestingly, despite using FeCl_3_ as the Fe precursor, the presence of Fe^2+^ indicates the partial reduction of Fe^3+^ during synthesis. This reduction can be attributed to the reducing environment created by dextrose during the green synthesis process, where the free aldehyde group of the reducing sugar is oxidized to the corresponding carboxylic acid while donating electrons that reduce a fraction of the Fe^3+^ to Fe^2+^ [25]. The mixed Fe-Ti NP exhibited the same qualitative behavior, with the Fe^3+^ 2p3/2 peak at 710.43 eV, satellite features at 718.91 and 723.67 eV, and a Fe^2+^ contribution at 726.37 eV, confirming that the mixed Fe^3+^/Fe^2+^ valence is preserved when Fe is incorporated into the Ti-rich framework [10].

The Ti2p region of the individual Ti NP exhibited a well-defined doublet structure characteristic of Ti^4+^ species. After calibration, the Ti2p3/2 peak at 458.41 eV shifted to 458.00 eV (68.6%) and the Ti2p1/2 peak at 464.12 eV shifted to 463.71 eV (31.4%), with a spin–orbit splitting of 5.71 eV and a doublet area ratio of approximately 2.18 to 1, close to the ideal 2:1 ratio expected for a 2p3/2 to 2p1/2 spin–orbit pair [30]. These binding energies align with previously reported values for Ti^4+^ in TiO_2_ [22]. No low-binding-energy shoulder was detected near 457 eV, where a reduced Ti^3+^ component would appear [31], confirming that Ti is present essentially exclusively as fully oxidized Ti^4+^ without detectable sub-stoichiometric reduction. The mixed Fe-Ti NP showed the corresponding Ti^4+^ doublet at 458.90 eV (13.04%) and 464.59 eV (6.26%) [10], indicating that the Ti oxidation state is unchanged upon combination with Fe and that Ti^4+^ forms the primary oxide framework of the mixed material.

The O1s spectrum was deconvoluted into two distinct O environments for each sample. For the Ti NP, lattice metal-oxide oxygen (M-O) was observed at 529.39 eV (80.8%), attributed to lattice oxygen, and surface hydroxyl, adsorbed, and defect oxygen at 530.83 eV (19.2%). For the Fe NP, the lattice Fe-O (M-O) component appeared at 530.54 eV (43.7%) and the surface hydroxyl and adsorbed oxygen component at 531.99 eV (56.3%). The mixed Fe-Ti NP showed lattice M-O oxygen at 530.18 eV (25.22%) and surface OH groups at 531.29 eV (20.45%) [10]. In all cases, the lower-binding-energy component corresponds to lattice oxygen and the higher-binding-energy component, located about 1 to 2 eV above it, corresponds to surface OH and adsorbed oxygen species, consistent with established assignments for metal oxide NPs [28]. The notably high hydroxyl fraction of the Fe NP surface (56.3%) contrasts with the lattice-oxygen-dominated Ti NP and indicates an extensively hydroxylated iron-oxide surface, beneficial for interfacial reactivity.

Taken together, the high-resolution XPS data reveals a coherent picture of the surface chemistry of the three materials. The individual Ti NP is a fully oxidized, stoichiometric, X-ray amorphous Ti^4+^ titania phase with a predominantly lattice-oxygen surface and no detectable Ti^3+^, whereas the individual Fe NP is a mixed-valence Fe^3+^/Fe^2+^ Fe oxide with an extensively hydroxylated surface and a significant burden of dextrose-derived organic C. In the mixed Fe-Ti NP, the dominant Ti^4+^ signal and high Ti:Fe ratio indicate that Ti forms the structural framework and contributes chemical and thermal stability, while the minor Fe component retains its mixed Fe^3+^/Fe^2+^ valence and its diagnostic Fe^3+^ shake-up satellite, confirming that the redox-active Fe centers survive incorporation into the titania-rich matrix [10]. These surface characteristics are directly relevant to the electrochemical and catalytic behavior of the materials. The abundant surface OH groups promote interfacial charge transfer and provide active sites for the adsorption and activation of reactant species [6,24], while the coexistence of Fe^3+^ and Fe^2+^ furnishes an intrinsic Fe^3+^/Fe^2+^ redox couple that can mediate electron transfer and activate the otherwise redox-inert Ti^4+^ framework [6,24]. The combination of a stable Ti^4+^ scaffold, mixed-valence Fe redox centers, and an OH-rich surface therefore provides a mechanistic basis for the enhanced electrochemical and catalytic efficiency of the mixed Fe-Ti NP relative to its individual Fe and Ti counterparts.

#### 2.1.4. X-Ray Diffraction Analysis

**Figure 9:**
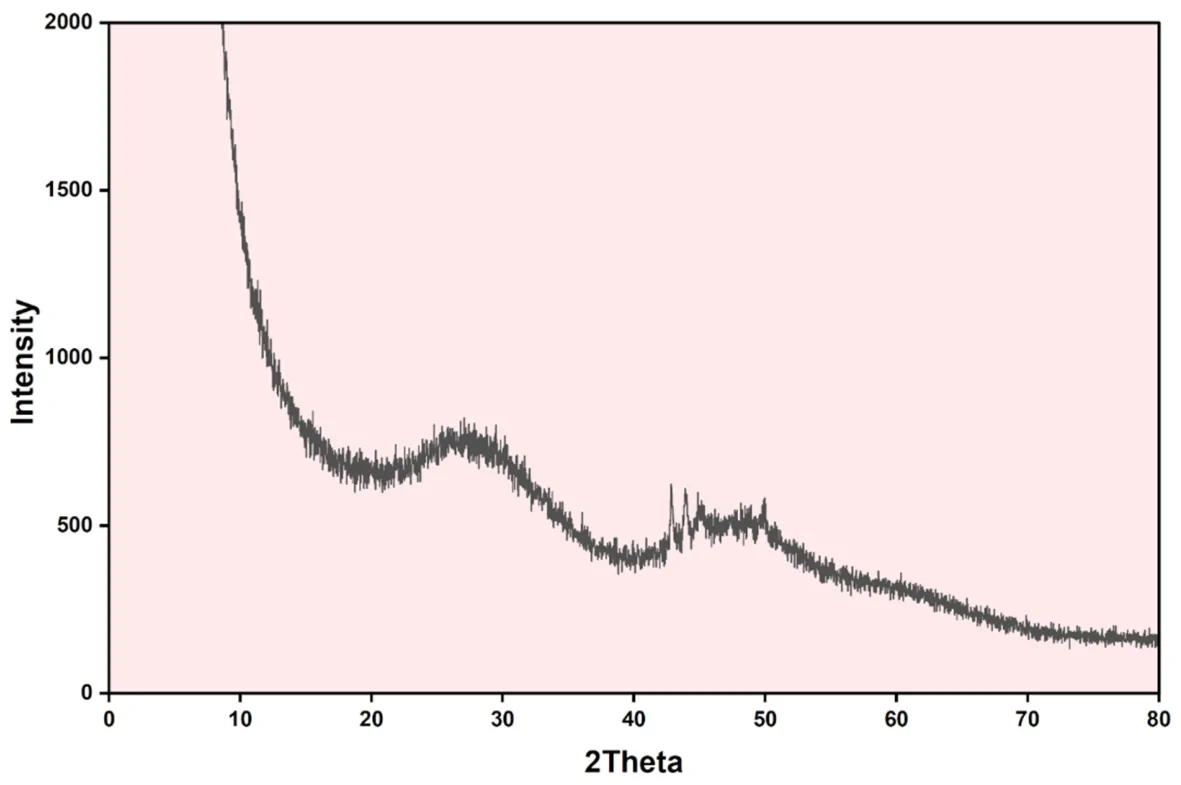
Powder X-ray diffraction pattern of the green-synthesized titanium nanoparticles (Ti NPs), recorded with Cu Kα radiation (λ = 1.5406 Å) over 0 to 80 degrees 2θ.

Figure 9 presents the powder XRD pattern of the green-synthesized Ti NPs. The diffractogram is essentially featureless, showing a steep increase in scattered intensity at very low angles below 10 degrees 2θ that decays rapidly, followed by a flat, low background on which only a single very broad and weak hump, centered near 27 to 30 degrees 2θ, can be discerned, together with barely perceptible undulations in the 43-to-50-degree region.

No sharp Bragg reflections are observed anywhere across the scanned range, and in particular there is no evidence of the principal reflection of crystalline anatase (near 25.3 degrees 2θ) or of rutile. The Ti NPs are therefore X-ray amorphous and lack long-range structural order. This result is fully consistent with the low-temperature, calcination-free green synthesis employed here, in which hydrolysis and condensation of Ti(IV) n-butoxide followed by drying at only 60 degrees Celsius are insufficient to nucleate a crystalline titania phase [17,32,33,34]. It is well established that titania derived from Ti alkoxides remains X-ray amorphous when dried at low temperature and that crystallization of the anatase polymorph requires calcination in the approximate range of 350 to 450 degrees Celsius, with significant anatase crystal growth appearing only above about 350 degrees Celsius [17,33,34]. The broad hump near 27 to 30 degrees reflects short-range order in the amorphous oxide and may also contain a contribution from residual amorphous carbon arising from the dextrose reducing agent, which typically gives a diffuse band in this angular region [34].

**Figure 10:**
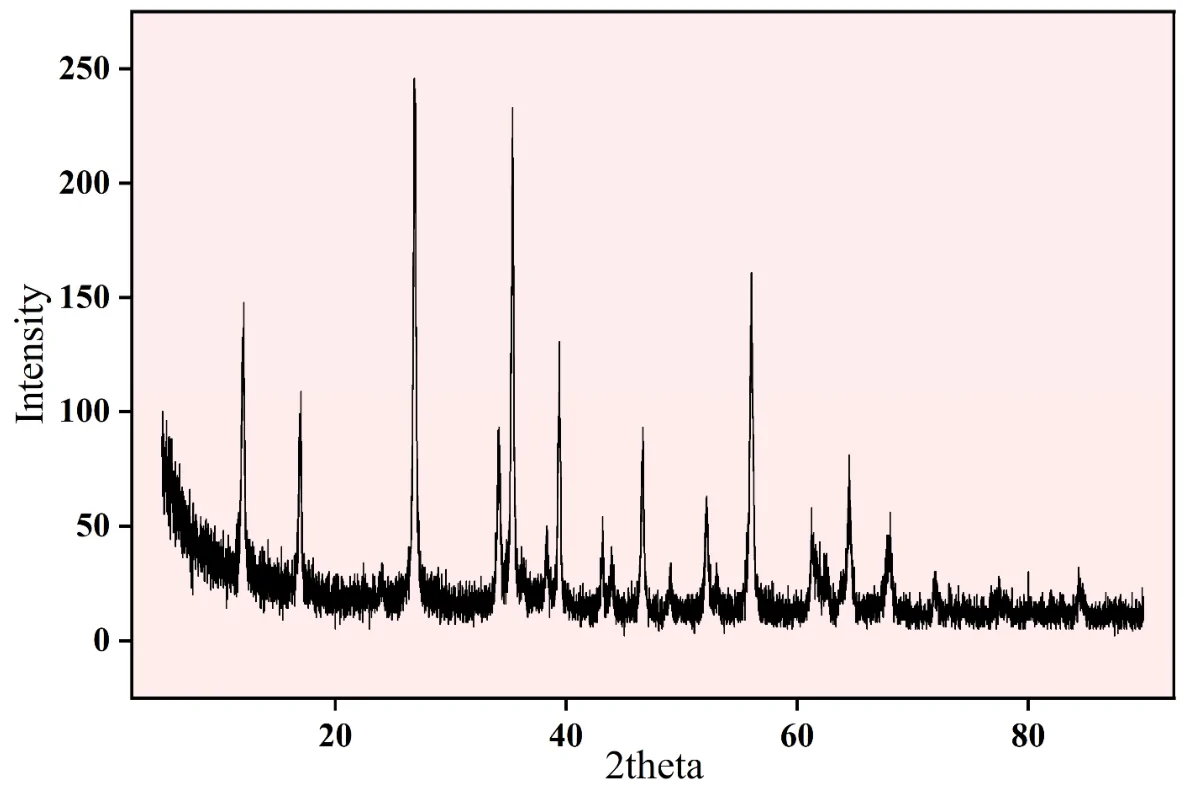
Powder X-ray diffraction pattern of the green-synthesized iron nanoparticles (Fe NPs), recorded with Cu Kα radiation (λ = 1.5406 Å) over 5 to 90 degrees 2θ.

In sharp contrast, the XRD pattern of the Fe NPs (Figure 10) is highly crystalline, exhibiting numerous sharp, well-defined reflections on a low background across 5 to 90 degrees 2θ.

The most prominent maxima appear near 12, 17, 27 (strongest), 34 to 35 (an intense, closely spaced pair), 39, 43 to 44, 47, 52, 56, 61, 64 and 68 degrees 2θ, with additional weaker reflections extending to about 85 degrees 2θ. The overall pattern indicates a well-ordered crystalline solid, but its phase assignment merits caution. In particular, the strong reflections at low angles near 12 and 17 degrees 2θ are not characteristic of the common simple Fe oxides, since magnetite (Fe_3_O_4_), maghemite (γ-Fe_2_O_3_) and hematite (α-Fe_2_O_3_) have their principal reflections above about 24 to 35 degrees 2θ and do not produce intense scattering near 12 and 17 degrees [35]. These low-angle reflections instead coincide closely with the (110) and (200) reflections of akaganeite (β-FeOOH), which are reported near 11.8 and 16.7 degrees 2θ, and whose further diagnostic reflections near 26.7, 34.1, 39.3, 46.5 and 55.8 degrees 2θ also fall close to features observed in the present pattern [36,37]. Akaganeite is a chloride-bearing Fe (III) oxyhydroxide with a tetragonal, hollandite-type tunnel structure (space group I4/m, a ≈ 10.48 Å, c ≈ 3.02 Å) whose approximately 0.5 by 0.5 nm tunnels are occupied and stabilized by chloride ions, and it forms preferentially by hydrolysis of chloride-rich Fe(III) solutions at low pH, that is, precisely from the FeCl_3_ precursor used here [20,37,38]. Given the calcination-free synthesis and the chloride-rich medium, the low-angle reflections are most plausibly attributed to akaganeite and/or to other residual, precursor-derived crystalline phases such as Fe oxychloride (FeOCl), which likewise displays an intense low-angle basal reflection near 11.2 degrees 2θ corresponding to an interlayer spacing of about 0.79 nm [9,39], or a hydrated Fe chloride. This interpretation is corroborated by the residual chlorine detected by XPS in these green-synthesized materials [10].

As reported previously for the same synthetic system, the mixed Fe-Ti material is X-ray amorphous, showing only a broad, diffuse peak centered at 25 to 30 degrees 2θ with no sharp crystalline reflections [10]. The mixed material therefore closely resembles the Ti NPs rather than the crystalline Fe NPs, indicating that in the mixed system the titania framework dominates the structure and suppresses crystallization of the Fe phase, an outcome consistent with the strongly Ti-rich composition (Ti to Fe atomic ratio of about 13 to 1) established by EDS and XPS for these samples [10]. In effect, dispersion of the minor Fe fraction within the amorphous titania network, combined with the mild drying conditions, prevents the segregation of the crystalline chloride-bearing Fe phase that is observed when Fe is synthesized alone. Taken together, the amorphous and defect-rich character of the Ti NPs and the mixed Fe-Ti NPs is favorable for the intended applications, since amorphous and poorly crystalline metal oxides generally expose more coordinatively unsaturated sites and larger accessible surface areas than their crystalline counterparts, features that are widely associated with enhanced adsorption and with improved electrochemical and catalytic activity [10,40].

### 2.2. Cyclic Voltammetry

**Figure 11:**
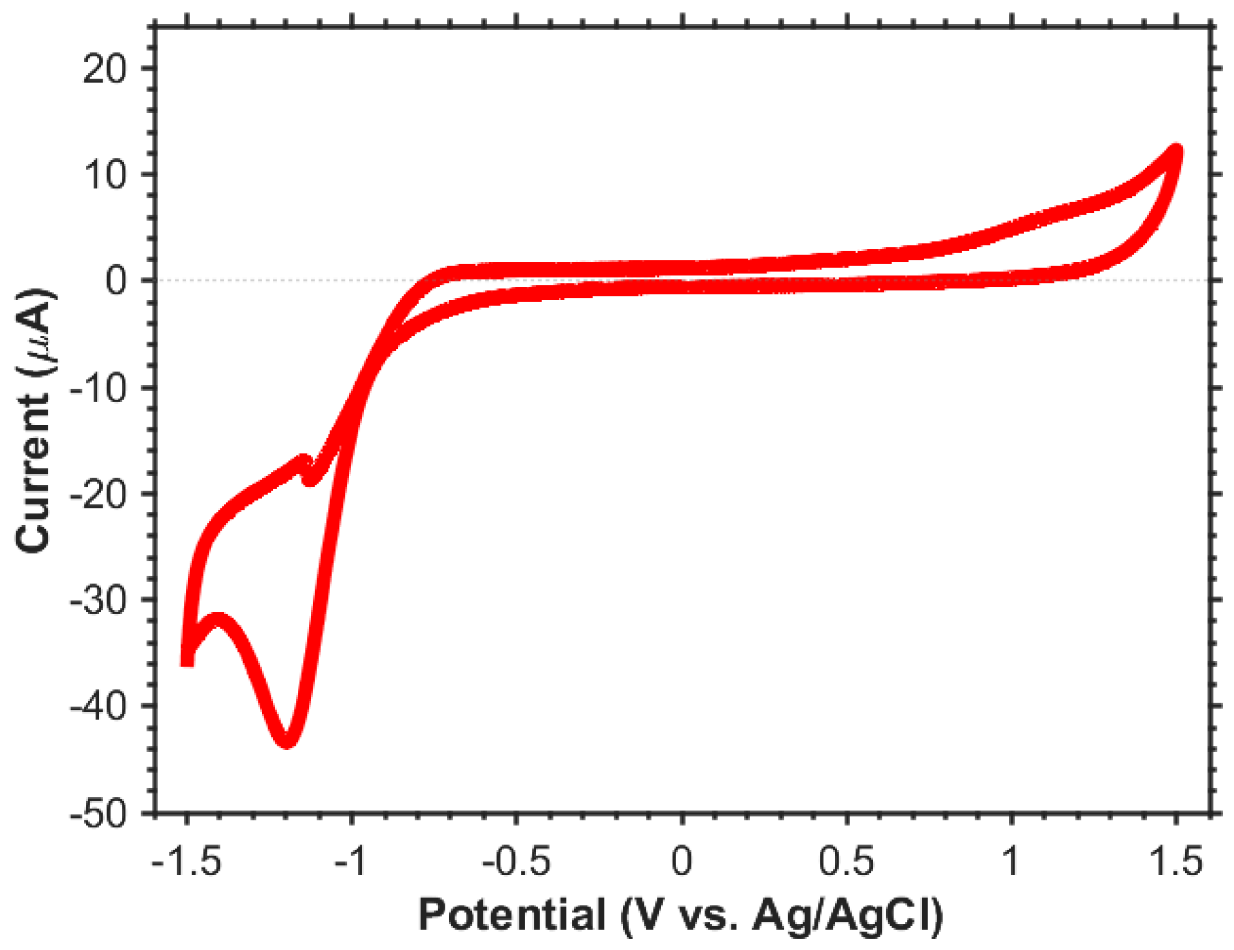
Cyclic voltammogram of the green-synthesized Fe NPs deposited on a carbon working electrode, recorded in 50 mM phosphate buffer (pH 7.4) between −1.5 V and +1.5 V versus Ag/AgCl at a scan rate of 10 mV/s.

**Figure 12:**
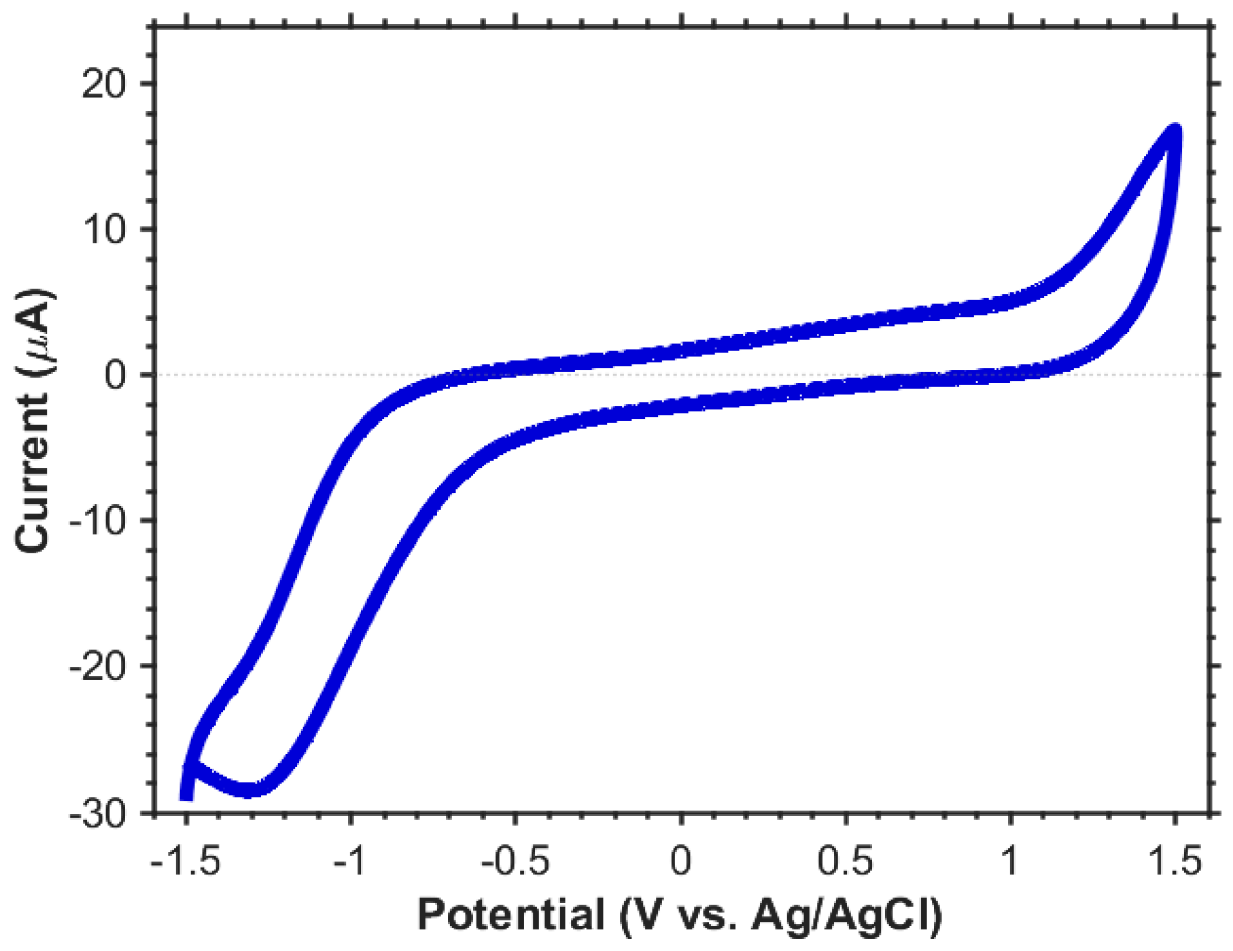
Cyclic voltammogram of the green-synthesized Ti NPs deposited on a carbon working electrode, recorded in 50 mM phosphate buffer (pH 7.4) between −1.5 V and +1.5 V versus Ag/AgCl at a scan rate of 10 mV/s.

**Figure 13:**
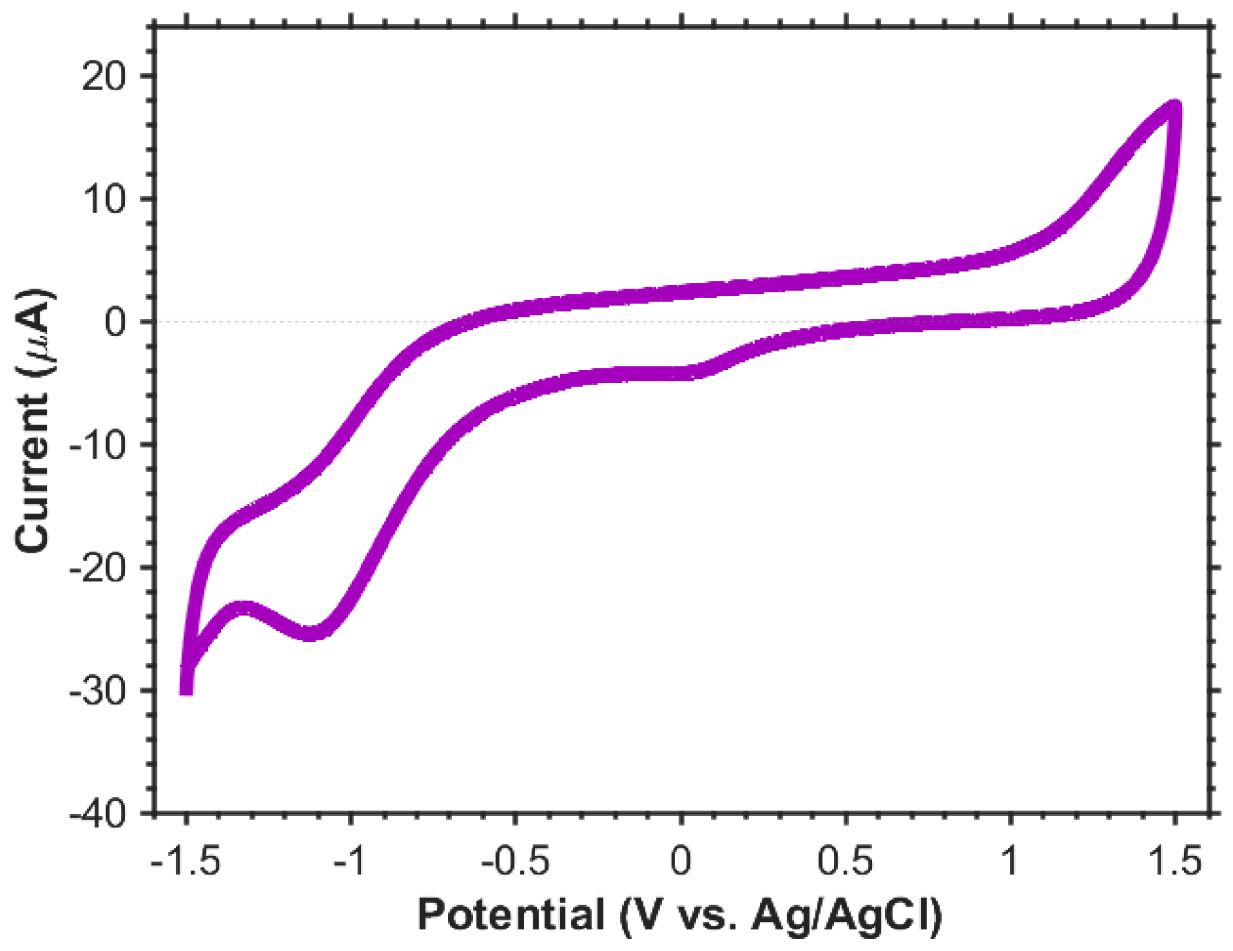
Cyclic voltammogram of the green-synthesized mixed Fe-Ti NPs deposited on a carbon working electrode, recorded in 50 mM phosphate buffer (pH 7.4) between −1.5 V and +1.5 V versus Ag/AgCl at a scan rate of 10 mV/s.

The electrochemical behavior of the three green-synthesized NP films was evaluated by CV in 50 mM phosphate buffer (pH 7.4) over the range −1.5 V to +1.5 V versus Ag/AgCl at 10 mV s^−1^. No external redox mediator was added to the electrolyte, so the recorded current originates from the deposited films together with the accessible solvent and electrolyte window. The voltammograms of the Fe, Ti, and mixed Fe-Ti NPs are presented in Figure 11, Figure 12 and Figure 13, respectively.

The Fe NP film (Figure 11) exhibited the most sharply defined faradaic response of the three materials. On the negative-going sweep, the cathodic current rose from an onset of approximately −0.85 V to a well-resolved peak at −1.199 V with a peak current of −43.4 μA. Beyond this maximum, the current decayed to a local minimum of −31.8 μA at −1.407 V and then increased once more in magnitude, reaching −34.3 μA at the negative limit. This sequence of a discrete peak followed by a current minimum and a renewed rise indicates two distinct cathodic processes operating in series rather than a single broad reduction.

The peak at −1.199 V is assigned to the reduction of surface Fe(III) centers to Fe(II) and related lower-valence species within the oxide film, consistent with the cathodic features reported for Fe oxide NPs supported on carbon electrodes in neutral and phosphate-containing media, where reduction in Fe(III) toward Fe(OH)_2_ and related phases occurs in this potential region [41]. The decay in current immediately following the peak is the signature of depletion of the reducible surface population, while the subsequent growth of cathodic current beyond −1.4 V is attributed to the onset of proton and water reduction, a process for which iron oxide films on carbon are known to be active in neutral phosphate electrolytes at these potentials [42].

The positive-going sweep of the Fe NP film contained no anodic peak of any magnitude; the current instead rose monotonically toward the positive limit and reached only +12.1 μA at +1.5 V, the smallest anodic response of the three materials. The complete absence of an oxidative counterpart establishes that the cathodic process is electrochemically irreversible under these conditions, behavior expected both for proton and water reduction, which have no matching anodic wave on the reverse scan, and for proton-coupled reduction in an Fe oxyhydroxide film [41,43].

The Ti NP film (Figure 12) presented a markedly more capacitive response. Through the central region of the window, the voltammogram adopted a broad, quasi-rectangular envelope characteristic of double-layer charging rather than of discrete faradaic turnover, and the separation between the forward and reverse sweeps in this region was appreciably wider than that of the Fe NP film, indicating a larger contribution from double-layer charging. Superimposed on this capacitive background was a single broad cathodic feature centered at −1.32 V with a current of −31.2 μA, beyond which the current decreased slightly in magnitude toward the negative limit. No anodic peak was observed on the return sweep, and the anodic current rose to +16.4 μA at +1.5 V. The breadth and poor definition of this cathodic feature, in contrast to the sharp peak of the Fe NP film, is characteristic of an amorphous, wide band gap Ti(IV) oxide (titania), which behaves as a semiconductor of low electronic conductivity and is essentially redox-inert through the mid potential range, so that its voltammetric signature is dominated by capacitive charging [44,45]. The feature is therefore assigned not to a discrete surface redox couple but to the onset of electron accumulation within the oxide, in which conduction band filling and the associated formation of Ti(III) and trap states become accessible only at sufficiently negative potentials, together with the concurrent onset of water and proton reduction. Nanostructured TiO_2_ electrodes in aqueous electrolyte are well documented to display precisely this accumulation behavior superimposed on a dominant capacitive envelope [44,46].

The mixed Fe-Ti NP film (Figure 13) displayed the largest overall electrochemical response and, uniquely among the three materials, combined resolved faradaic features with an extended capacitive envelope. Three cathodic features were distinguished on the negative-going sweep: a small but well-defined peak at −0.041 V (−4.4 μA), a principal broad peak at −1.13 V (−25.5 μA), and a renewed increase in cathodic current to −28.4 μA at the negative limit following a local minimum near −1.33 V. The anodic current reached +17.1 μA at +1.5 V, the highest of the three films, and once again no oxidation peak was resolved on the return sweep. The principal cathodic feature is assigned to reduction in the Fe component, demonstrating that the Fe redox chemistry remains electroactive when dispersed within the Ti-rich matrix. Its broadening and diminished intensity relative to the pure Fe NP film are consistent with Fe sites distributed throughout the mixed oxide rather than concentrated as a discrete Fe oxide phase, which produces a wider distribution of site energies and hence a broader wave. Worth noting is the small peak resolved near 0 V, which has no counterpart in the Ti NP voltammogram and is not resolved in the pure Fe NP film. Its appearance indicates an additional, readily accessible surface-confined Fe redox process that is expressed only in the mixed material, and it is this feature that most directly signals a change in the electrochemical environment of the Fe centers upon combination with the Ti oxide framework [41].

Comparison of the three voltammograms reveals systematic trends that place the mixed Fe-Ti NPs ahead of single-component materials. The enclosed loop area, evaluated over an identical potential cycle, increased in the order of 14.20 microamp volts for the Fe NPs, 18.85 for the Ti NPs, and 22.20 for the mixed Fe-Ti NPs. The enclosed area of a voltammogram is widely used as a qualitative indicator of the electrochemically active surface area and charge-storage capability of metal oxide NP films [47]. On this basis, the mixed material presents the largest accessible electroactive interface. More informative mechanistically, the mixed Fe-Ti film also exhibited the narrowest polarization gap of the three. Cathodic current became appreciable at −0.36 V, compared with −0.56 V for the Ti NPs and −0.85 V for the Fe NPs, while the anodic current likewise rose earliest, at +0.93 V compared with +0.99 V and +1.00 V. Both electrode reactions therefore commence at lower overpotential on the mixed material than on either constituent. Because the enclosed area and onset potentials are also influenced by the electroactive surface area and by capacitive contributions, these observations are interpreted as an enhanced overall electrochemical response rather than as direct evidence of faster interfacial charge transfer, which would require impedance measurements to establish.

This combination of properties is noteworthy. The Fe NP film delivers an intense but highly localized and irreversible faradaic peak together with the smallest capacitive envelope and the smallest anodic current, whereas the Ti NP film offers a broad capacitive envelope, but little resolved faradaic chemistry. Only the mixed material expresses both, and it does so while reducing the overpotential required for the cathodic and anodic processes alike. Such behavior parallels reports that incorporation of Fe into a titania framework improves the electrical conductivity of the otherwise redox-inert oxide and lowers the interfacial charge-transfer resistance, while the resulting Fe(III)/Fe(II) couple contributes pseudo-capacitance in addition to the double-layer response [6,48], although confirming a lowered charge-transfer resistance for the present material would require impedance spectroscopy. The larger anodic currents recorded for the Ti and Fe-Ti films relative to the Fe film are attributed to the onset of water oxidation on the more extensive oxide surface, consistent with the earlier anodic onset measured for the mixed material.

### 2.3. MO Degradation

**Figure 14:**
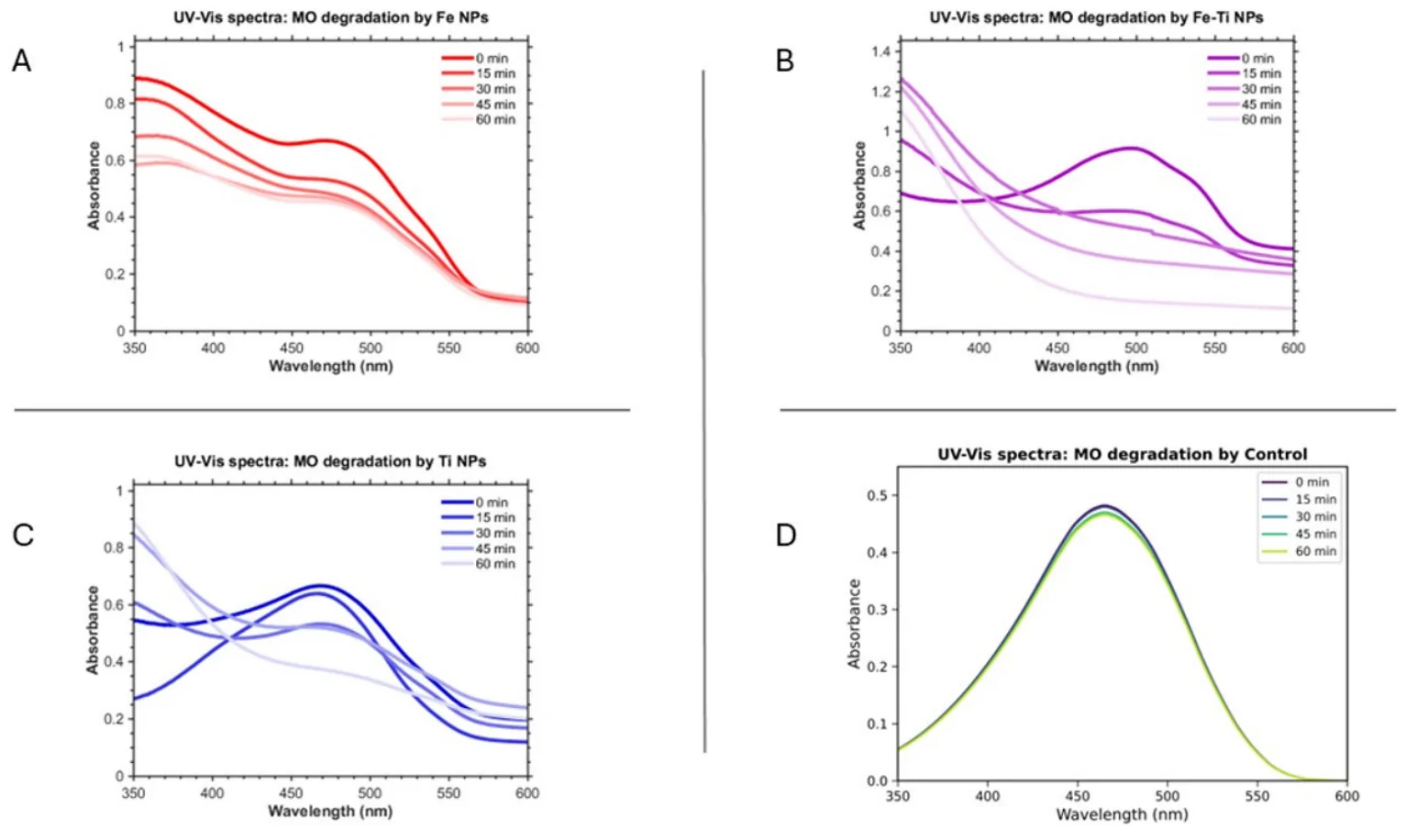
UV-Vis absorption spectra (350 to 600 nm) showing the degradation of methyl orange over 0, 15, 30, 45, and 60 min in the presence of (**A**) Fe NPs, (**B**) mixed Fe-Ti NPs, (**C**) Ti NPs, and (**D**) the control (no catalyst).

**Figure 15:**
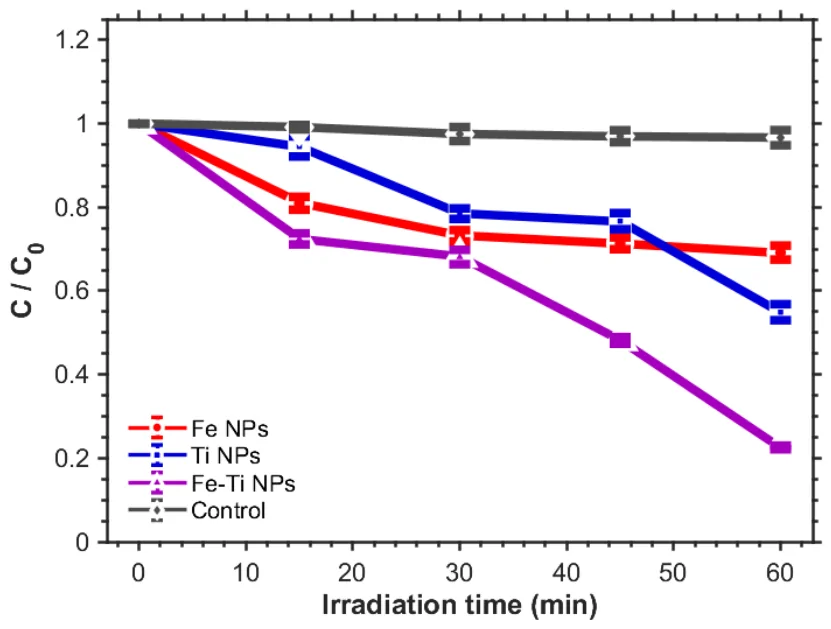
Normalized MO concentration (C/C_0_) as a function of reaction time for the Fe NPs, Ti NPs, mixed Fe-Ti NPs, and control (mean plus or minus SD, n = 3).

**Figure 16:**
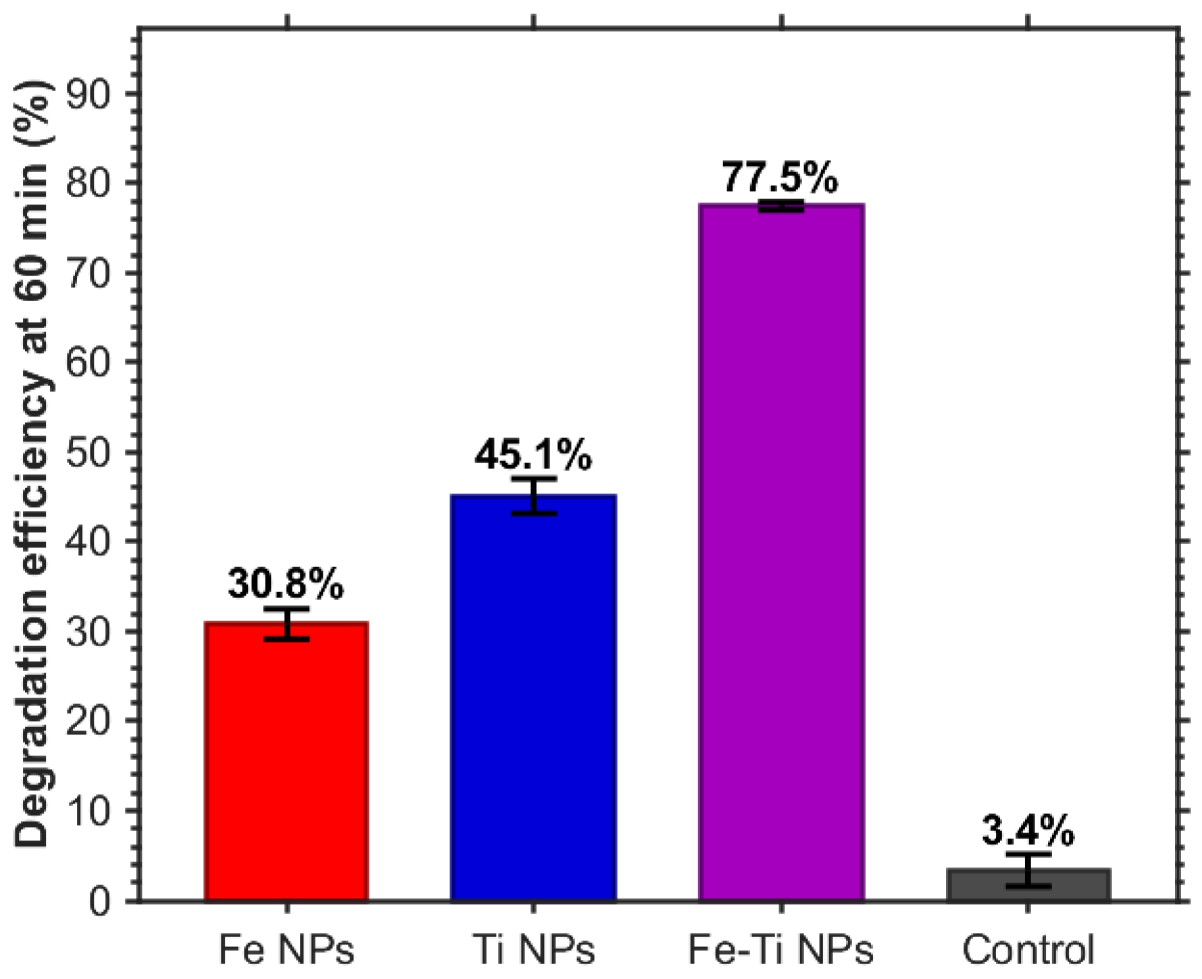
MO degradation efficiency for the Fe NPs, Ti NPs, mixed Fe-Ti NPs, and control (mean plus or minus SD, n = 3).

The catalytic activity of the green-synthesized Fe, Ti, and mixed Fe-Ti NPs was evaluated by monitoring the degradation of MO, a model azo dye, in the presence of H_2_O_2_, following an approach similar to that reported previously for milk-derived Fe NPs [49]. In a typical experiment, 2 mg of the NP catalyst was added to a 50 mL beaker containing 8.9 mL of distilled water, 100 microliters of MO solution (2.25 mM), and 1 mL of 10% H_2_O_2_, giving a total reaction volume of 10 mL, a catalyst loading of 0.2 g L−1, an initial MO concentration of approximately 0.0225 mM, and a final H_2_O_2_ concentration of approximately 0.29 M. The three catalysts were compared at an identical total catalyst mass rather than at matched metal content or matched surface area. On the basis of the EDS-derived metal fractions, this corresponds to approximately 0.76 mg of Fe for the Fe NPs and approximately 1.23 mg of Ti for the Ti NPs, with the mixed material being strongly Ti-dominant and containing only a minor Fe fraction. Because the three materials differ in composition, and because specific surface areas were not measured in this study, the activities reported below are compared on an equal-mass basis, and normalization to metal content or surface area was not attempted. The corresponding oxidant-to-dye molar ratio was therefore large, on the order of 1.3 × 10^4^ mol H_2_O_2_ per mol MO, so that H_2_O_2_ was present in substantial excess and was not rate-limiting during the measurements. The mixture was stirred continuously with a magnetic stirrer at room temperature under ambient laboratory light, without a dedicated illumination or UV source, and its UV-Vis absorption spectrum was recorded at intervals over the 350 to 600 nm range until the characteristic orange color of the dye had dissipated. The intensity of the MO absorption maximum near 465 nm was used to follow the dye concentration as a function of time. A control experiment was performed under identical conditions, including the same H_2_O_2_ addition, but without any NP catalyst, in order to distinguish the activity of the NPs from any degradation caused by H_2_O_2_ alone. All experiments were carried out in triplicate, and the results are reported as mean plus or minus one standard deviation. The decolorization efficiency, taken as a measure of dye removal based on the loss of the 465 nm absorbance, was calculated as (A_0_ − A_t_)/A_0_ × 100, where A_0_ is the absorbance at the MO maximum at the start of the reaction and A_t_ is the absorbance at time t. The catalytic performance of the three green-synthesized materials was evaluated through the degradation of MO in the presence of H_2_O_2_, a heterogeneous Fenton-like process in which the NPs activate H_2_O_2_ to generate reactive OH radicals that oxidize the dye [6,50]. The evolution of the UV-Vis absorption spectra, the normalized concentration profiles (C/C_0_), and the degradation efficiencies are presented in Figure 14, Figure 15 and Figure 16, respectively.

The UV-Vis spectra of the treated MO solutions showed a progressive decrease in the intensity of the characteristic azo-dye absorption band near 465 nm with increasing reaction time, confirming the destruction of the chromophore. In the presence of the Fe NPs, the band diminished steadily over the reaction period, while the Ti NPs and the mixed Fe-Ti NPs produced a more pronounced decrease accompanied by changes in the overall spectral profile, including altered absorbance at shorter wavelengths that is indicative of the formation of degradation intermediates during the transformation of the dye rather than simple decolorization. In contrast, the control experiment, which contained H_2_O_2_ but no NPs (Figure 14D), showed only a negligible change in the MO band, confirming that H_2_O_2_ alone does not appreciably degrade the dye and that the observed removal is catalyst-driven. It should be emphasized that the loss of the 465 nm band measures decolorization through destruction of the azo chromophore, and that the accompanying growth of shorter-wavelength features indicates chemical transformation of the dye rather than physical adsorption or simple bleaching. UV-Vis monitoring alone, however, does not quantify the extent of molecular degradation or mineralization, which would require complementary total organic carbon (TOC) analysis together with high-pressure liquid chromatography or liquid chromatography mass spectrometry, and these measurements were not carried out in the present study. A dedicated adsorption control, in which each catalyst is contacted with the dye in the absence of H_2_O_2_, was likewise not included, so a minor adsorptive contribution to the observed removal cannot be fully excluded. Adsorption is nonetheless expected to be limited under the present conditions, because the anionic MO dye and the negatively charged catalyst surface at near-neutral pH (the mixed material has a low point of zero charge of 0.22 [8]) are electrostatically repulsive, so the removal is attributed predominantly to oxidative transformation.

The normalized concentration profiles (Figure 15) quantified these differences. The control retained essentially all of its initial dye concentration, with C/C_0_ remaining close to unity throughout. The Fe NPs reduced C/C_0_ to approximately 0.69, and the Ti NPs, following an initial period during which little change occurred, reduced C/C_0_ to approximately 0.53. The mixed Fe-Ti NPs exhibited the most rapid and sustained decline, with C/C_0_ decreasing steadily to approximately 0.22. The corresponding degradation efficiencies were 3.4% for the control, 30.8% for the Fe NPs, 45.1% for the Ti NPs, and 77.5% for the mixed Fe-Ti NPs. The mixed material therefore degraded substantially more dye than either single-component oxide, and its efficiency exceeded the combined contributions of the individual Fe and Ti NPs. We describe this as an enhanced rather than a strictly additive activity, and we note that establishing a genuine interfacial synergy, as distinct from compositional and surface-area effects, would require comparison with a physically mixed Fe and Ti control of matched Fe:Ti ratio, which was not included in the present study.

The decolorization profiles were further analyzed using a pseudo-first-order kinetic model, and the resulting apparent rate constants, coefficients of determination, and initial rates are summarized in Table 2. The apparent rate constant of the mixed Fe-Ti NPs (20.6 × 10^−3^ min^−1^) was approximately 2.8 times that of the Fe NPs and 2.5 times that of the Ti NPs, consistent with the decolorization efficiencies. Because the profiles deviate somewhat from ideal first-order behavior for some samples, these values are reported as apparent rate constants that provide a consistent basis for comparing the relative reactivity of the catalysts rather than as evidence for a specific rate law.

The superior performance of the mixed Fe-Ti NPs is consistent with the behavior widely reported for combined Fe-Ti oxide catalysts. In the proposed Fenton-like pathway, the Fe centers are expected to be the principal sites for activation of H_2_O_2_ into OH radicals, while coupling the Fe phase with the titania framework is thought to promote charge separation and to provide interfacial electron-transfer pathways that support Fe^3+^/Fe^2+^ redox cycling and sustained radical generation, in contrast to the more limited redox turnover of the single oxides [6].

This mechanism is proposed on the basis of the material characterization and measured activity trends rather than demonstrated directly, since radical-trapping and electron paramagnetic resonance experiments were not performed in this study. The comparatively modest activity of the individual Fe and Ti NPs is in line with this interpretation, since neither component alone provides the combined radical-generating and charge-separating functionality of the mixed material.

The efficiency achieved by the green-synthesized mixed Fe-Ti NPs compares favorably with values reported in the literature for related Fe-Ti oxide systems evaluated against MO. A green-combustion-synthesized TiO_2_-Fe_2_O_3_ nanocomposite was likewise found to decolorize MO more effectively than its constituent TiO_2_ and Fe_2_O_3_ nanoparticles, mirroring the enhancement observed here [6]. A TiO_2_/beta-FeOOH heterostructured catalyst was reported to exhibit markedly higher MO degradation than either pure beta-FeOOH or TiO_2_ in a heterogeneous Fenton-like process, an observation of particular relevance given the Fe oxyhydroxide character of the present Fe component [51]. Taken together, these comparisons indicate that the mixed Fe-Ti NPs synthesized here by a simple, low-temperature dextrose-mediated green route deliver catalytic performance competitive with, and in several cases superior to, that of comparable Fe-Ti oxide catalysts prepared by more energy-intensive methods. It should be noted that the present degradation experiments were conducted under a large excess of H_2_O_2_, chosen to ensure that the oxidant was not rate-limiting when comparing the three catalysts. For practical wastewater treatment, the H_2_O_2_ dose would need to be optimized, since excess peroxide adds cost and can itself scavenge OH radicals. A systematic evaluation of the H_2_O_2_ concentration dependence and of oxidant consumption and utilization efficiency was outside the scope of this study and represents an important direction for future work.

## 3. Discussion

The central finding of this study is that the green-synthesized mixed Fe-Ti NPs outperform both single-component materials simultaneously in electrochemistry and in catalysis, delivering the largest cyclic voltammetric response together with the highest Fenton-like MO degradation (77.5% at 60 min, versus 45.1% for Ti NPs, 30.8% for Fe NPs, and 3.4% for the catalyst-free control). Both behaviors are attributable to the same underlying feature, an accessible Fe^3+^/Fe^2+^ redox couple associated with a minor, well-dispersed Fe component on a high-surface-area amorphous titania framework.

The three surface and structural techniques converge on a consistent identity for each phase. The Fe component is not a simple Fe oxide but a crystalline, chloride-bearing, mixed-valence Fe (oxyhydr)oxide whose diffraction, vibrational, and surface signatures are most consistent with an akaganeite-type (β-FeOOH) phase, with possible contributions from other precursor-derived chloride-containing phases such as Fe oxychloride (FeOCl). XPS placed the Fe 2p_3_/_2_ envelope at 710.97 eV with the diagnostic Fe^3+^ satellite near 717.98 eV and an extensively hydroxylated surface, XRD showed the anomalous low-angle reflections near 12° and 17° 2θ characteristic of the chloride-filled β-FeOOH tunnel structure, and FTIR resolved the akaganeite Fe-O-H and Fe-O bands at 843 and 653 cm^−1^, while the residual chloride found by EDS (4.11 at%) is itself a hallmark of this phase [18,19,28]. The nanoscale character of the materials is supported indirectly by the crystalline akaganeite phase, which characteristically grows as nanoscale domains, and by the X-ray amorphous, low-order character of the titania and mixed phases, together with the fine particulate morphology seen by SEM. Direct imaging of individual particles by transmission electron microscopy (TEM) was not performed in this study, and TEM together with a complementary sizing method such as dynamic light scattering is recommended to confirm the primary particle dimensions. The Ti component, by contrast, is an amorphous, OH-bearing but stoichiometric Ti^4+^ titania, showing only Ti^4+^ by XPS, no crystalline reflections by XRD, and a broad Ti-O-Ti envelope near 595 to 631 cm^−1^, a material expected to be largely redox-inert under aqueous polarization [44]. In the mixed material the amorphous, high-surface-area titania framework (BET 292.89 m^2^ g^−1^ reported previously [10]) dominates the architecture and disperses the minor Fe fraction (Ti:Fe ≈ 13:1) as an accessible, redox-active Fe component rather than allowing it to grow into the bulk akaganeite crystals seen in the pure Fe sample. It should be noted that the present techniques (XPS, XRD, and EDS) establish that the Fe is minor, mixed-valence, and highly dispersed, but they do not distinguish isolated Fe sites incorporated into the titania from small, dispersed Fe (oxyhydr)oxide (FeOx/FeOOH) domains. Resolving the two would require local probes such as high-resolution transmission electron microscopy or X-ray absorption spectroscopy. Such amorphous, defect-rich oxides are widely reported to expose more coordinatively unsaturated active sites than their crystalline analogues [52].

This architecture is expressed directly in electrochemistry. The Ti NPs gave only a broad capacitive envelope with no genuine redox couple, consistent with a redox-inert semiconductor, whereas the Fe NPs showed a sharp but irreversible Fe^3+^ to Fe^2+^ cathodic peak at −1.199 V. Despite containing far less Fe, the Fe-Ti NPs produced the largest enclosed voltammetric loop area (22.20 µA·V, versus 18.85 for Ti and 14.20 for Fe) and the narrowest polarization gap (cathodic onset −0.36 V, versus −0.56 and −0.85 V), signaling the largest overall electrochemical response of the three materials. The same trend, in which a mixed Ti-Fe oxide surpasses both single oxides, was reported for a TiO_2_-Fe_2_O_3_ nanocomposite that showed higher capacitance and the lowest charge-transfer resistance of the series, with well-defined redox peaks absent in the single oxides [6]. Fe Incorporation therefore confers accessible redox chemistry on the otherwise inert titania. The cyclic voltammetry establishes that the Fe^3+^/Fe^2+^ centers are redox-accessible and become more so in the mixed architecture, which is consistent with a Fenton-like mechanism in which the same couple activates H_2_O_2_. We note, however, that the voltammetry was recorded under an externally applied potential in phosphate buffer, whereas the dye degradation proceeds without applied polarization in a different medium, so the electrochemical measurements support the plausibility of Fe^3+^/Fe^2+^ participation rather than directly demonstrating this cycling under catalytic conditions. With that caveat, the electrochemical ranking parallels the catalytic ranking. In heterogeneous Fenton chemistry, surface Fe^2+^ activates H_2_O_2_ to release the OH radical that degrades the dye, and the regeneration of Fe^2+^ from Fe^3+^ is the slow, rate-limiting step [53,54]. Any feature that raises the population of accessible Fe sites or eases the electron transfer that reduces Fe^3+^ back to Fe^2+^ therefore increases the OH radical flux, and the mixed architecture does both, exactly the properties read from its largest CV loop area and narrowest polarization gap. The titania here plays the constructive role documented for titania-supported Fe Fenton catalysts, dispersing ultrafine iron, supplying surface OHs, and easing the interfacial charge migration that sustains Fe^3+^/Fe^2+^ cycling [55]. The degradation ranking, Fe-Ti (77.5%) > Ti (45.1%) > Fe (30.8%) > control (3.4%), thus follows the accessible redox chemistry rather than the bulk Fe content, and the mixed material shows enhanced degradation relative to the single components, consistent with the improved performance reported for TiO_2_-Fe_2_O_3_ and TiO_2_/β-FeOOH systems [6,55]. The surface OHs and dextrose-derived carboxylate groups identified by XPS and FTIR reinforce this by binding and activating H_2_O_2_ and by helping to shuttle the Fe^3+^/Fe^2+^ cycle at near-neutral pH [54], and the 77.5% conversion achieved thermally at near-neutral pH with a modest 0.2 g L^−1^ loading is competitive with green Fe-Ti systems that typically require UV or acidic conditions [6]. Finally, the recyclability and structural stability of the catalyst were not evaluated, and the concentrations of dissolved Fe and Ti in the treated solution were not measured, so a contribution from leached Fe acting as a homogeneous Fenton catalyst cannot be strictly excluded. Such a contribution is expected to be limited under the near-neutral conditions used here, since Fe leaching from Fe (oxyhydr)oxides is far more pronounced at acidic pH, but confirming a predominantly heterogeneous pathway would require measurement of metal leaching by ICP together with a hot-filtration test in which the catalyst is removed mid-reaction and the filtrate monitored for continued degradation. These stability, leaching, and reuse measurements are identified as essential steps for future work. Further consideration concerns the residual chloride retained in the Fe phase, which is a structural constituent of the akaganeite tunnels and originates from the FeCl_3_ precursor. During reaction, this chloride may be partially released into solution, which could in turn affect the stability of the chloride-bearing iron phase over repeated use. Chloride can also participate in oxidation chemistry, since chloride ions can scavenge OH radicals to form less reactive Cl-based radical species and can promote the formation of chlorinated organic intermediates, although chloride-bearing Fe phases such as Fe oxychloride are themselves known to be active Fenton-like catalysts. From an environmental standpoint, the release of chloride at the low levels present here is not expected to be of concern in itself, but the possible formation of chlorinated byproducts would need to be evaluated before practical application. Chloride release during reaction and the speciation of the degradation products were not measured in this study, and their assessment, together with the metal-leaching measurements noted above, is identified as an important direction for future work.

## 4. Materials and Methods

### 4.1. Chemicals and Materials

High-purity chemicals were purchased from Sigma-Aldrich (St. Louis, MO, USA) and used as received. Iron (III) chloride (FeCl_3_), tetra-butyl titanate, dextrose, ethanol, H_2_O_2_ (10%), and MO were used for synthesis and degradation experiments. All aqueous solutions were prepared with distilled water.

### 4.2. Synthesis of Nanoparticles

All three NP types were prepared by green synthesis using dextrose as the reducing agent. Full procedural details for the mixed Fe–Ti NPs were reported previously [10] and are summarized below.

#### 4.2.1. Iron Nanoparticles

Iron NPs were prepared following the method described by Gautam et al., with minor modifications [12]. Two volumes of pasteurized dextrose (0.4%) were mixed with 0.1 M FeCl_3_ solution (2:1 *v*/*v* sugar:FeCl_3_). The mixture was heated at 70 °C with continuous stirring on a magnetic stirrer for 15 min until the pale-yellow color changed to orange. The mixture was centrifuged at 15,000 rpm for 10 min, and the precipitate was washed with distilled water (4–5 times). After centrifugation, the collected precipitate was dried at 60 °C for 3 h and stored until characterization and dye degradation studies were performed.

#### 4.2.2. Titanium Nanoparticles

Tetra-butyl titanate (23 mL) was mixed with ethanol (23 mL) and stirred for 2 min to ensure homogenization. Dextrose (0.4%) was then introduced dropwise under continuous stirring until a white gel formed, indicating successful NP synthesis. The gel was transferred to a container and dried in an oven at 60 °C overnight to obtain the final dried NPs.

#### 4.2.3. Mixed Fe–Ti Nanoparticles

Tetra-butyl titanate (20 mL) was mixed with ethanol (20 mL) and stirred for 2 min. FeCl_3_ solution (40 mL, 0.1 M in ethanol) was then added gradually under stirring, which continued for a further 5 min. Dextrose (0.4%) was introduced under stirring until a yellow gel formed. The gel was dried as described for the Ti NP [8]. Table 3 lists the comparison of the synthesis methods of all NPs.

### 4.3. Characterizations

FTIR spectra were collected on a Thermo Fisher Scientific Nicolet iS5 FT-IR spectrometer (Thermo Scientific, Waltham, MA, USA) with OMNIC 9.13 software. UV-Vis measurements were performed on a Jasco V-770 spectrophotometer (Easton, MD, USA). XRD patterns were obtained using a 1 kW Cu X-ray tube operated at 40 kV and 25 mA. XPS spectra were acquired on a PHI Genesis spectrometer (Chanhassen, MN, USA). SEM and EDS analyses were performed on a JEOL JSM-6010LA (Tokyo, Japan) scanning electron microscope using InTouchScope software (version 1.11); samples were mounted on carbon-coated adhesive tape, and backscattered electron images were collected at an accelerating voltage of 10 kV, a beam current of ≈90 µA, and a working distance of 9 mm. EDS spectra were collected as area scans over an analyzed area of 0.15 mm^2^ (110 µm × 135 µm) for each sample, so that the reported compositions represent an area-averaged signal rather than a single-point interaction volume.

### 4.4. Cyclic Voltammetry Measurements

CV experiments on the NPs were performed using a CHI760E electrochemical workstation (CH Instruments, Austin, TX, USA), following an approach similar to the work previously reported for the electrochemical characterization of green-synthesized iron NPs [50]. A conventional three-electrode configuration was employed, consisting of an NP-deposited carbon working electrode, an Ag/AgCl reference electrode, and a platinum counter electrode. The reference electrode was an Ag/AgCl electrode in 3 M KCl, and the counter electrode was a platinum electrode. The working electrode was a glassy carbon electrode of 3 mm diameter, corresponding to a geometric area of approximately 0.071 cm^2^, which was polished, rinsed, and dried before use to provide a clean and reproducible surface. Working electrodes were prepared by drop-casting 5 µL of the NP suspension onto the electrode surface with Nafion as the binder and allowing the film to dry in air. A separate electrode was prepared for each material, and three independent electrodes were measured per material.

All measurements were carried out in 50 mM phosphate buffer (pH 7.4) as the supporting electrolyte at room temperature. No external redox probe was added, so the measured current arises from the deposited NP films together with the accessible electrolyte and solvent window. Voltammograms were recorded from an initial potential of 0 V, scanning first in the negative direction to a low potential limit of −1.5 V, then to a high potential limit of +1.5 V versus Ag/AgCl, at a scan rate of 10 mV/S. Data were acquired with a sample interval of 1 mV following a quiet time of 2 s, at a current sensitivity of 1 × 10^−3^ A/V. Two complete cycles were recorded for the Fe and Ti NPs, and the data from the second cycle are reported, while the mixed Fe-Ti NPs were recorded over three segments.

### 4.5. Dye Degradation Studies

NPs (2 mg) were added to a 50 mL beaker together with 8.9 mL of distilled water and 100 µL of MO solution (2.25 mM), followed by 1 mL of 10% H_2_O_2_ [12]. The degradation experiments were carried out in unbuffered distilled water at room temperature and at the natural near-neutral pH of the reaction mixture, with no acid, base, or buffer added. The mixture was stirred continuously with a magnetic stirrer under ambient laboratory light, without a dedicated illumination or UV source. At each time point, an aliquot was withdrawn and centrifuged, and the clear supernatant was collected to remove suspended nanoparticles before analysis. UV-Vis spectra were recorded over the 350 to 600 nm range in a 1 cm path length quartz cuvette, and the absorbance of the MO maximum at 465 nm was used to follow the dye concentration.

## 5. Conclusions

Three green-synthesized nanomaterials were prepared by a dextrose-reduced, low-temperature, calcination-free route and compared across structural, electrochemical, and catalytic measurements. XPS, XRD, and FTIR together identify the Fe component as a crystalline, mixed-valence, chloride-bearing Fe (oxyhydr)oxide most consistent with an akaganeite-type (β-FeOOH) phase, with possible contributions from other precursor-derived chloride phases such as FeOCl, and the Ti component as an amorphous, redox-inert Ti^4+^ titania, while in the mixed material the high-surface-area amorphous titania framework disperses a minor, redox-active Fe (oxyhydr)oxide component that retains accessible Fe^3+^/Fe^2+^ valence. This architecture gave the mixed Fe-Ti NPs the largest cyclic voltammetric loop area (22.20 µA·V) and the narrowest polarization gap, and the highest MO degradation (77.5% in 60 min, versus 45.1%, 30.8%, and 3.4% for Ti, Fe, and the control). The two performance domains are correlated: the accessible Fe^3+^/Fe^2+^ redox chemistry that produces the superior voltammetric response is also the chemistry expected to drive the Fenton-like activation of H_2_O_2_ responsible for dye degradation. Because the voltammetry was recorded under applied potential in phosphate buffer rather than under the open-circuit catalytic conditions, this link is presented as a mechanistically consistent correlation rather than a direct demonstration of Fe^3+^/Fe^2+^ cycling during catalysis. These findings show that a simple, mild, dextrose-mediated synthesis can yield a bifunctional Fe-Ti nanomaterial for water remediation. Future work should extend the CV to multiple scan rates, employ electron paramagnetic resonance and radical-scavenging experiments to identify the reactive oxygen species directly, and evaluate recyclability, stability, and metal leaching before testing against real wastewater.

## Figures and Tables

**Table 1:** Synthesis comparison of Fe-Ti, Fe and Ti nanoparticles.

Material	Synthesis Method	Precursors	Conditions	Key Outcome/Application	Reference
Mixed Fe–Ti oxide NPs	Green, dextrose-mediated (this work)	FeCl_3_, titanium(IV) n-butoxide, dextrose	60 °C drying, no calcination	Amorphous, chloride-bearing, high surface area; MO decolorization and CV	[10]
Composite Fe–Ti oxide NPs	Aerosol-assisted CVD	Fe(II) chloride, Ti oxyacetylacetonate	High-temperature gas phase	Crystalline Fe–Ti oxide; simultaneous As, Cd, Cu, Pb, F removal	[14]
Iron oxide–TiO_2_ composite NPs	Gas-phase spray pyrolysis	Fe and Ti aerosol precursors	High-temperature flame/spray	Encapsulated Fe-oxide–TiO_2_ composite NPs	[15]
TiO_2_–Fe_2_O_3_ nanocomposite	Green combustion (Aloe vera gel)	Ti and Fe nitrates, Aloe vera gel	Combustion and calcination	Crystalline TiO_2_–Fe_2_O_3_; dye decolorization, supercapacitor	[6]
Fe-doped TiO_2_ NPs	Sol–gel	Ti alkoxide, Fe salt	Calcination	Anatase Fe-doped TiO_2_; optical/photoluminescence	[16]
Carbon-containing TiO_2_ NPs	Sol–gel	Ti alkoxide	Calcination	Anatase titania; photocatalysis	[17]
Iron oxyhydroxide (akaganeite, β-FeOOH)	Forced hydrolysis of FeCl_3_	FeCl_3_ solution	Thermal hydrolysis, low pH	Chloride-bearing β-FeOOH	[18]

**Table 2:** Apparent pseudo-first-order kinetic parameters for the decolorization of MO by the green-synthesized Fe, Ti, and mixed Fe–Ti NPs and the catalyst-free control.

Catalyst	Kapp (×10^−3^ min^−1^)	R^2^	r_0_ (µM min^−1^)
Mixed Fe–Ti NPs	20.6 ± 2.4	0.88	0.42
Ti NPs	8.2 ± 1.0	0.87	0.08
Fe NPs	7.3 ± 0.9	0.74	0.29
Control (no catalyst)	0.6 ± 0.05	0.92	0.01

**Table 3:** Comparison of the synthesis conditions for the green-synthesized Fe, Ti, and mixed Fe–Ti nanomaterials. The three procedures differ in metal precursor, solvent and water content, and reaction conditions.

Parameter	Fe NPs	Ti NPs	Mixed Fe–Ti NPs
Metal precursor	0.1 M FeCl_3_	Tetra-butyl titanate (23 mL)	Tetra-butyl titanate (20 mL) + 0.1 M FeCl_3_ (40 mL)
Solvent/medium	Aqueous (distilled water)	Ethanol (23 mL)	Ethanol (FeCl_3_ dissolved in ethanol)
Reducing agent	Dextrose 0.4% (2:1 *v*/*v* sugar:FeCl_3_)	Dextrose 0.4% (added dropwise)	Dextrose 0.4%
Reaction conditions	70 °C, 15 min, stirred	Room temp, ~2 min stir, gel formation	Room temp, ~5 min stir, gel formation
Product form	Orange precipitate	White gel	Yellow gel
Work-up	Centrifuged (15,000 rpm, 10 min), washed 4–5× with distilled water	No washing step	No washing step
Drying	60 °C, 3 h	60 °C, overnight	60 °C, overnight

## Data Availability

All data are available within the manuscript. Further inquiries can be directed to the corresponding author.

## Abbreviations

The following abbreviations are used in this manuscript:

Fe	Iron
Ti	Titanium
Cu	Copper
C	Carbon
O	Oxygen
Cl	Chlorine
OH	Hydroxyl
FeCl_3_	Fe(III)Chloride
NP	Nanoparticle
FTIR	Fourier-transform infrared spectroscopy
XPS	X-ray photoelectron spectroscopy
XRD	X-ray diffraction
UV-Vis	Ultraviolet and visible
MO	Methyl orange
AOP	Advanced oxidation process
H_2_O_2_	Hydrogen peroxide
CV	Cyclic voltammetry

## References

[1] Al-Tohamy R., Ali S.S., Li F., Okasha K.M., Mahmoud Y.A.-G., Elsamahy T., Jiao H., Fu Y., Sun J. (2022). A critical review on the treatment of dye-containing wastewater: Ecotoxicological and health concerns of textile dyes and possible remediation approaches for environmental safety. Ecotoxicol. Environ. Saf..

[2] Berradi M., Hsissou R., Khudhair M., Assouag M., Cherkaoui O., El Bachiri A., El Harfi A. (2019). Textile finishing dyes and their impact on aquatic environs. Heliyon.

[3] Thomas N., Dionysiou D.D., Pillai S.C. (2021). Heterogeneous Fenton catalysts: A review of recent advances. J. Hazard. Mater..

[4] Chen C., Xu F., Tan Z., Fei Y., Wang L., Hao D., Du H., Wang Q. (2026). The three-electron oxygen reduction reaction for hydroxyl radical generation: Mechanistic insights and catalysts design for environmental applications. Chem. Eng. J..

[5] Jin L., Gao J., Lin Y., Wu Y., Zhu H., Wang Q. (2026). Design of Cu-Doped Goethite for Fenton-like Degradation of Tetracycline: An Integrative Undergraduate Laboratory Module in Wastewater Remediation. J. Chem. Educ..

[6] Kumar M.A., Abebe B., Nagaswarupa H., Murthy H.A., Ravikumar C., Sabir F.K. (2020). Enhanced photocatalytic and electrochemical performance of TiO_2_-Fe_2_O_3_ nanocomposite: Its applications in dye decolorization and as supercapacitors. Sci. Rep..

[7] Ismael M. (2020). Enhanced photocatalytic hydrogen production and degradation of organic pollutants from Fe (III) doped TiO_2_ nanoparticles. J. Environ. Chem. Eng..

[8] Yang S., Yang L., Liu X., Xie J., Zhang X., Yu B., Wu R., Li H., Chen L., Liu J. (2015). TiO_2_-doped Fe_3_O_4_ nanoparticles as high-performance Fenton-like catalyst for dye decoloration. Sci. China Technol. Sci..

[9] Raveendran P., Fu J., Wallen S.L. (2003). Completely “green” synthesis and stabilization of metal nanoparticles. J. Am. Chem. Soc..

[10] Hewage S.P., Fernando H. (2025). Green Synthesis and Characterization of Fe-Ti Mixed Nanoparticles for Enhanced Lead Removal from Aqueous Solutions. Molecules.

[11] Li M., Song J., Han W., Yeung K.L., Zhou S., Mo C.-H. (2023). Iron-organic frameworks as effective fenton-like catalysts for peroxymonosulfate decomposition in advanced oxidation processes. npj Clean Water.

[12] Lv X., Leng Y., Wang R., Wei Y., Ren X., Guo W. (2022). Persulfate activation by ferrocene-based metal–organic framework microspheres for efficient oxidation of orange acid 7. Environ. Sci. Pollut. Res..

[13] Akerdi A.G., Es’haghzade Z., Bahrami S., Arami M. (2017). Comparative study of GO and reduced GO coated graphite electrodes for decolorization of acidic and basic dyes from aqueous solutions through heterogeneous electro-Fenton process. J. Environ. Chem. Eng..

[14] Monárrez-Cordero B., Sáenz-Trevizo A., Bautista-Carrillo L., Silva-Vidaurri L., Miki-Yoshida M., Amézaga-Madrid P. (2018). Simultaneous and fast removal of As^3+^, As^5+^, Cd^2+^, Cu^2+^, Pb^2+^ and F^−^ from water with composite Fe-Ti oxides nanoparticles. J. Alloys Compd..

[15] Harra J., Nikkanen J.-P., Aromaa M., Suhonen H., Honkanen M., Salminen T., Heinonen S., Levänen E., Mäkelä J. (2013). Gas phase synthesis of encapsulated iron oxide–titanium dioxide composite nanoparticles by spray pyrolysis. Powder Technol..

[16] Husain S., Alkhtaby L.A., Giorgetti E., Zoppi A., Miranda M.M. (2016). Investigation of the role of iron doping on the structural, optical and photoluminescence properties of sol–gel derived TiO_2_ nanoparticles. J. Lumin..

[17] Moussa I., Ibrahim H., Emam E.-A.M., Tawfik T.M. (2022). Structure, light absorption properties and photocatalytic activity of carbon-containing titania nanocomposites synthesized via a facile sol–gel method. Heliyon.

[18] Musić S., Krehula S., Popović S., Skoko Ž. (2003). Some factors influencing forced hydrolysis of FeCl_3_ solutions. Mater. Lett..

[19] Murad E., Bishop J.L. (2000). The infrared spectrum of synthetic akaganéite, β-FeOOH. Am. Mineral..

[20] Cai J., Liu J., Gao Z., Navrotsky A., Suib S.L. (2001). Synthesis and Anion Exchange of Tunnel Structure Akaganeite. Chem. Mater..

[21] Jubb A.M., Allen H.C. (2010). Vibrational spectroscopic characterization of hematite, maghemite, and magnetite thin films produced by vapor deposition. ACS Appl. Mater. Interfaces.

[22] León A., Reuquen P., Garín C., Segura R., Vargas P., Zapata P., Orihuela P.A. (2017). FTIR and Raman characterization of TiO_2_ nanoparticles coated with polyethylene glycol as carrier for 2-methoxyestradiol. Appl. Sci..

[23] Rojas J., Toro-Gonzalez M., Molina-Higgins M., Castano C. (2016). Facile radiolytic synthesis of ruthenium nanoparticles on graphene oxide and carbon nanotubes. Mater. Sci. Eng. B.

[24] Li T.-T., Shi B.-Y., Jiang L.-W., Zheng J.-F., Wang J.-J. (2022). Design and preparation of NiFe_2_O_4_@FeOOH composite electrocatalyst for highly efficient and stable oxygen evolution reaction. Molecules.

[25] Granata G., Onoguchi A., Tokoro C. (2019). Preparation of copper nanoparticles for metal-metal bonding by aqueous reduction with d-glucose and PVP. Chem. Eng. Sci..

[26] Grosvenor A., Kobe B., Biesinger M.C., McIntyre N. (2004). Investigation of multiplet splitting of Fe 2p XPS spectra and bonding in iron compounds. Surf. Interface Anal..

[27] Yao B., Luo Z., Du S., Yang J., Zhi D., Zhou Y. (2021). Sustainable biochar/MgFe_2_O_4_ adsorbent for levofloxacin removal: Adsorption performances and mechanisms. Bioresour. Technol..

[28] Biesinger M.C., Payne B.P., Grosvenor A.P., Lau L.W., Gerson A.R., Smart R.S.C. (2011). Resolving surface chemical states in XPS analysis of first row transition metals, oxides and hydroxides: Cr, Mn, Fe, Co and Ni. Appl. Surf. Sci..

[29] Yamashita T., Hayes P. (2008). Analysis of XPS spectra of Fe^2+^ and Fe^3+^ ions in oxide materials. Appl. Surf. Sci..

[30] Biesinger M.C., Lau L.W., Gerson A.R., Smart R.S.C. (2010). Resolving surface chemical states in XPS analysis of first row transition metals, oxides and hydroxides: Sc, Ti, V, Cu and Zn. Appl. Surf. Sci..

[31] Nikam R.D., Kwak M., Hwang H. (2021). All-solid-state oxygen ion electrochemical random-access memory for neuromorphic computing. Adv. Electron. Mater..

[32] Cho W., Lee S.-H., Ju W.-S., Baek Y., Lee J.K. (2004). Conversion of natural gas to hydrogen and carbon black by plasma and application of plasma carbon black. Catal. Today.

[33] Ohtani B., Ogawa Y., Nishimoto S.-I. (1997). Photocatalytic activity of amorphous− anatase mixture of titanium (IV) oxide particles suspended in aqueous solutions. J. Phys. Chem. B.

[34] Sevilla M., Fuertes A.B. (2009). The production of carbon materials by hydrothermal carbonization of cellulose. Carbon.

[35] Saif S., Tahir A., Chen Y. (2016). Green Synthesis of Iron Nanoparticles and Their Environmental Applications and Implications. Nanomaterials.

[36] Song X., Boily J.-F. (2011). Surface Hydroxyl Identity and Reactivity in Akaganéite. J. Phys. Chem. C.

[37] Post J.E., Buchwald V.F. (1991). Crystal structure refinement of akaganéite. Am. Mineral..

[38] Scheck J., Lemke T., Gebauer D. (2015). The Role of Chloride Ions during the Formation of Akaganéite Revisited. Minerals.

[39] Yang X.-J., Xu X.-M., Xu J., Han Y.-F. (2013). Iron Oxychloride (FeOCl): An Efficient Fenton-Like Catalyst for Producing Hydroxyl Radicals in Degradation of Organic Contaminants. J. Am. Chem. Soc..

[40] Smith R.D.L., Prévot M.S., Fagan R.D., Zhang Z., Sedach P.A., Siu M.K.J., Trudel S., Berlinguette C.P. (2013). Photochemical Route for Accessing Amorphous Metal Oxide Materials for Water Oxidation Catalysis. Science.

[41] Corsini M., Fabrizi de Biani F., Atrei A. (2024). Electrochemical properties of magnetite nanoparticles supported on carbon paste electrode in various electrolyte solutions: A study by cyclic voltammetry. J. Appl. Electrochem..

[42] Ibarra J., Aguirre M.J., Del Río R., Henriquez R., Faccio R., Dalchiele E.A., Arce R., Ramírez G. (2024). A-Fe_2_O_3_/, Co_3_O_4_/, and CoFe_2_O_4_/MWCNTs/ionic liquid nanocomposites as high-performance electrocatalysts for the electrocatalytic hydrogen evolution reaction in a neutral medium. Int. J. Mol. Sci..

[43] Aeppli M., Voegelin A., Gorski C.A., Hofstetter T.B., Sander M. (2018). Mediated electrochemical reduction of iron (oxyhydr-) oxides under defined thermodynamic boundary conditions. Environ. Sci. Technol..

[44] Fabregat-Santiago F., Mora-Seró I., Garcia-Belmonte G., Bisquert J. (2003). Cyclic voltammetry studies of nanoporous semiconductors. Capacitive and reactive properties of nanocrystalline TiO_2_ electrodes in aqueous electrolyte. J. Phys. Chem. B.

[45] Kavan L. (2024). Electrochemistry and band structure of semiconductors (TiO_2_, SnO_2_, ZnO): Avoiding pitfalls and textbook errors. J. Solid State Electrochem..

[46] Kamisan A., Kudin T., Ali A., Yahya M. (2011). Electrical and physical studies on 49% methyl-grafted natural rubber-based composite polymer gel electrolytes. Electrochim. Acta.

[47] Sun S., Lang J., Wang R., Kong L., Li X., Yan X. (2014). Identifying pseudocapacitance of Fe_2_O_3_ in an ionic liquid and its application in asymmetric supercapacitors. J. Mater. Chem. A.

[48] George S., Pokhrel S., Ji Z., Henderson B.L., Xia T., Li L., Zink J.I., Nel A.E., Mädler L. (2011). Role of Fe doping in tuning the band gap of TiO_2_ for the photo-oxidation-induced cytotoxicity paradigm. J. Am. Chem. Soc..

[49] Gautam I., Grady T., Fernando H. (2023). Degradation of the dye methyl orange using cow and goat milk iron nanoparticles. Green Chem. Lett. Rev..

[50] Buchadas A., Vaz A.S., Honrado J.P., Alagador D., Bastos R., Cabral J.A., Santos M., Vicente J.R. (2017). Dynamic models in research and management of biological invasions. J. Environ. Manag..

[51] Xu Z., Zhang M., Wu J., Liang J., Zhou L., Lǚ B. (2013). Visible light-degradation of azo dye methyl orange using TiO_2_/β-FeOOH as a heterogeneous photo-Fenton-like catalyst. Water Sci. Technol..

[52] Park J., Lee S., Kim S. (2022). Recent advances in amorphous electrocatalysts for oxygen evolution reaction. Front. Chem..

[53] Deng F., Olvera-Vargas H., Zhou M., Qiu S., Sirés I., Brillas E. (2023). Critical review on the mechanisms of Fe^2+^ regeneration in the electro-Fenton process: Fundamentals and boosting strategies. Chem. Rev..

[54] Yan Q., Zhang J., Xing M. (2020). Cocatalytic Fenton reaction for pollutant control. Cell Rep. Phys. Sci..

[55] Guadagnini A., Agnoli S., Badocco D., Pastore P., Coral D., van Raap M.B.F., Forrer D., Amendola V. (2021). Facile synthesis by laser ablation in liquid of nonequilibrium cobalt-silver nanoparticles with magnetic and plasmonic properties. J. Colloid Interface Sci..

